# Vitamin C Can Shorten the Length of Stay in the ICU: A Meta-Analysis

**DOI:** 10.3390/nu11040708

**Published:** 2019-03-27

**Authors:** Harri Hemilä, Elizabeth Chalker

**Affiliations:** 1 Department of Public Health, University of Helsinki, POB 41, FI-00014 Helsinki, Finland; 2 School of Public Health, University of Sydney, Sydney 2006, Australia; elizabeth.chalker@gmail.com

**Keywords:** antioxidants, burns, artificial respiration, cardiac surgical procedures, cardiovascular system, critical care, dietary supplements, oxidative stress, sepsis, systematic review

## Abstract

A number of controlled trials have previously found that in some contexts, vitamin C can have beneficial effects on blood pressure, infections, bronchoconstriction, atrial fibrillation, and acute kidney injury. However, the practical significance of these effects is not clear. The purpose of this meta-analysis was to evaluate whether vitamin C has an effect on the practical outcomes: length of stay in the intensive care unit (ICU) and duration of mechanical ventilation. We identified 18 relevant controlled trials with a total of 2004 patients, 13 of which investigated patients undergoing elective cardiac surgery. We carried out the meta-analysis using the inverse variance, fixed effect options, using the ratio of means scale. In 12 trials with 1766 patients, vitamin C reduced the length of ICU stay on average by 7.8% (95% CI: 4.2% to 11.2%; *p* = 0.00003). In six trials, orally administered vitamin C in doses of 1–3 g/day (weighted mean 2.0 g/day) reduced the length of ICU stay by 8.6% (*p* = 0.003). In three trials in which patients needed mechanical ventilation for over 24 hours, vitamin C shortened the duration of mechanical ventilation by 18.2% (95% CI 7.7% to 27%; *p* = 0.001). Given the insignificant cost of vitamin C, even an 8% reduction in ICU stay is worth exploring. The effects of vitamin C on ICU patients should be investigated in more detail.

## 1. Introduction

For many centuries, scurvy was a severe disease most commonly afflicting sailors on long voyages [1,2,3,4,5,6,7,8,9,10,11,12,13,14,15,16]. In 1753, James Lind, a Scottish surgeon, reported that scurvy could be successfully treated with citrus fruit. However, it was not until the 1930s that Albert Szent-Györgyi discovered that vitamin C was the specific cure for scurvy [17]. Since then, it seems that many people have also believed the converse to be true; that is, that scurvy is the only disease for which vitamin C is effective. This history has led to a widespread belief that vitamin C is only useful for the prevention and treatment of scurvy. However, there is strong evidence that vitamin C is also effective for many conditions other than scurvy.

In controlled trials, vitamin C has improved endothelial function [18,19,20], lowered blood pressure [21], increased left ventricular ejection fraction [22,23,24,25,26], decreased the incidence of atrial fibrillation [27,28,29], protected against contrast-induced acute kidney injury [30,31], decreased glucose levels in patients with type 2 diabetes [32], decreased bronchoconstriction [33,34,35], shortened the duration of colds [36,37,38,39,40,41,42], decreased the incidence of colds in physically stressed people [41,42,43], and it has prevented pain [44,45,46,47]. There is also evidence that vitamin C may have a beneficial effect on pneumonia [2,42,48,49]. These findings demonstrate that the physiological effects of vitamin C are not limited to preventing and treating scurvy, although conclusions about the practical significance of these effects require further exploration.

In experimental vitamin C deficiency studies, scurvy is induced when dietary vitamin C intake and plasma vitamin C levels are below approximately 10 mg/day and 11 μmol/L, respectively [50,51,52,53,54,55,56,57,58,59,60,61,62]. In the USA, the average vitamin C intake is about 100 mg/day [63] and this leads to plasma levels of 50–60 μmol/L [62]. These are important reference levels when considering vitamin C doses and plasma levels in intensive care unit (ICU) patients.

While scurvy is relatively rare nowadays, it has not disappeared altogether. A number of case reports have described patients with overt scurvy in diverse hospital settings, including ICUs [13,64,65,66,67,68,69,70,71,72,73,74,75,76,77,78,79,80,81]. Furthermore, even though clinical scurvy is rare, very low vitamin C plasma levels, such as those found in scurvy patients, are not uncommon in hospitals. Several studies have been undertaken looking at this issue [82,83,84,85,86,87,88,89].

A survey of elderly Scottish patients hospitalized because of acute respiratory infections reported that 35% of patients had vitamin C plasma levels less than 11 μmol/L [82]. One study in a hospital in Paris reported that 44% of patients had vitamin C plasma levels less than 6 μmol/L [84], and in another hospital in Paris, 17% of patients had levels less than 11 μmol/L [85]. Another survey, of patients with advanced cancer in a hospice, found that 30% had vitamin C plasma levels less than 11 μmol/L [86]. In a Canadian university hospital, it was found that 19% of patients had vitamin C plasma levels less than 11 μmol/L [87]. In a study of surgical patients in Australia, it was found that 21% had vitamin C plasma levels less than 11 μmol/L [89]. Finally, in another study, 3% of cancer patients had clinical scurvy and vitamin C plasma levels less than 6 μmol/L [83], and in a survey of 145 hospitalized elderly French patients, 18 had clinical symptoms of scurvy [88]. Although these estimates of prevalence for vitamin C deficiency should not be extrapolated directly to other hospital settings, they demonstrate that low vitamin C levels are common in some contexts.

Given this, it would seem reasonable to screen plasma vitamin C levels in hospital patients when appropriate and administer vitamin C to patients with particularly low levels, irrespective of whether vitamin C is believed to be effective for conditions other than scurvy.

Furthermore, there is evidence that vitamin C metabolism is changed in many conditions that involve physiological stress, such as infections, surgery, traumas, and burns, in which case vitamin C levels can decline dramatically [90,91,92,93,94,95,96,97,98,99,100,101]. Although 0.1 g/day of vitamin C can maintain a normal plasma level in a healthy person [62], much higher doses (1–4 g/day) are needed for critically ill patients to increase plasma vitamin C levels to the normal range [102,103,104,105]. Therefore, high vitamin C doses may be needed to compensate for the increased metabolism to reach normal plasma levels.

Given that vitamin C has diverse effects on medical conditions [18,19,20,21,22,23,24,25,26,27,28,29,30,31,32,33,34,35,36,37,38,39,40,41,42,43,44,45,46,47,48,49], it is possible that, in some contexts, these effects may also lead to practical benefits. A previous meta-analysis of 11 trials on patients undergoing elective cardiac surgery found that vitamin C shortened hospital stay on average by 10% [28]. The purpose of this current meta-analysis was to analyze controlled trials which have reported on the effect of vitamin C on the length of ICU stay or on the duration of mechanical ventilation.

## 2. Methods

### 2.1. Selection Criteria for Trials and the Searches

We included controlled trials that compared the length of ICU stay and/or the duration of mechanical ventilation between vitamin C and control groups. We included trials in which the administration of vitamin C was the only difference between the study groups, so that trials which administered other therapies as well as vitamin C were included, if the other therapies were the same for both trial groups. We did not limit our search to randomized trials and we did not require placebo control. We included all doses and all durations of vitamin C administration.

**Figure 1:**
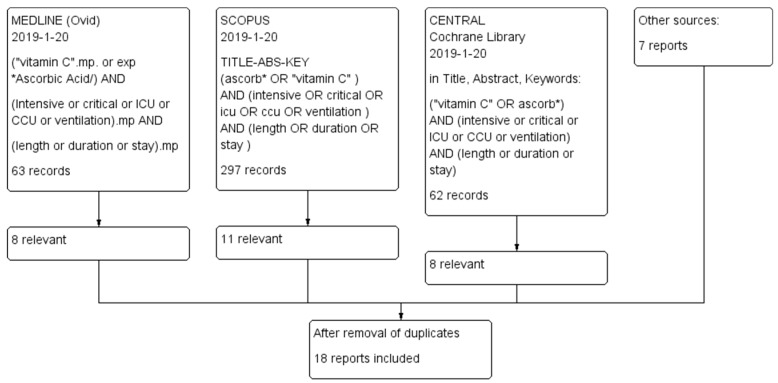
Flow diagram of the searches. The search terms and the number of identified records are shown in this figure. One of the identified trials recruited 500 participants, but reported only 290 participants [123]. This is such a great violation of the intention-to-treat (ITT) principle that we excluded that study from our analyses. One publication reported two separate trials [122]. This leads to the 18 included trials from the 18 identified publications.

We searched MEDLINE (Ovid), Scopus and the Cochrane Register (CENTRAL; Cochrane Library) from their inception to 20 January 2019. We did not impose any restrictions on the language of publication. The search strategies are detailed in Figure 1. In addition, we checked the reference lists of the included trials, closely related papers and relevant reviews. We identified 18 publications in all [106,107,108,109,110,111,112,113,114,115,116,117,118,119,120,121,122,123] (Table 1; Table S1 of Supplementary file S1). One publication reported a 2 × 2 factorial trial on vitamin C and vitamin E [122]. In our analysis, this trial was split into two trials: those receiving vitamin E and those not receiving vitamin E. One study started with 500 participants, but reported the findings for just 290 participants [123] (Table S1). That is such a severe violation of the intention-to-treat (ITT) principle [124,125,126] that we excluded that study from further analyses altogether. Thus, we included 18 controlled trials in our analysis.

### 2.2. Outcomes

The primary outcome of interest in our study was length of ICU stay, and the secondary outcome was duration of mechanical ventilation. Moreover, we collected data for tertiary outcomes from the included trials when they appeared relevant for this review. These included multi-organ failure scores, inflammation markers, dose and duration of norepinephrine administration, creatine kinase MB isoenzyme (CK-MB) levels, requirement for fluid infusions, and cardiac index during follow-up (see Results).

### 2.3. Selection of Studies and Data Extraction

Both authors independently screened the titles and abstracts and identified trials for inclusion. One author (HH) extracted study characteristics and outcomes from the trials and entered the data in a spreadsheet. Both authors checked the data entered against the original trial reports.

### 2.4. Quality Assessment of the Trials

**Figure 2:**
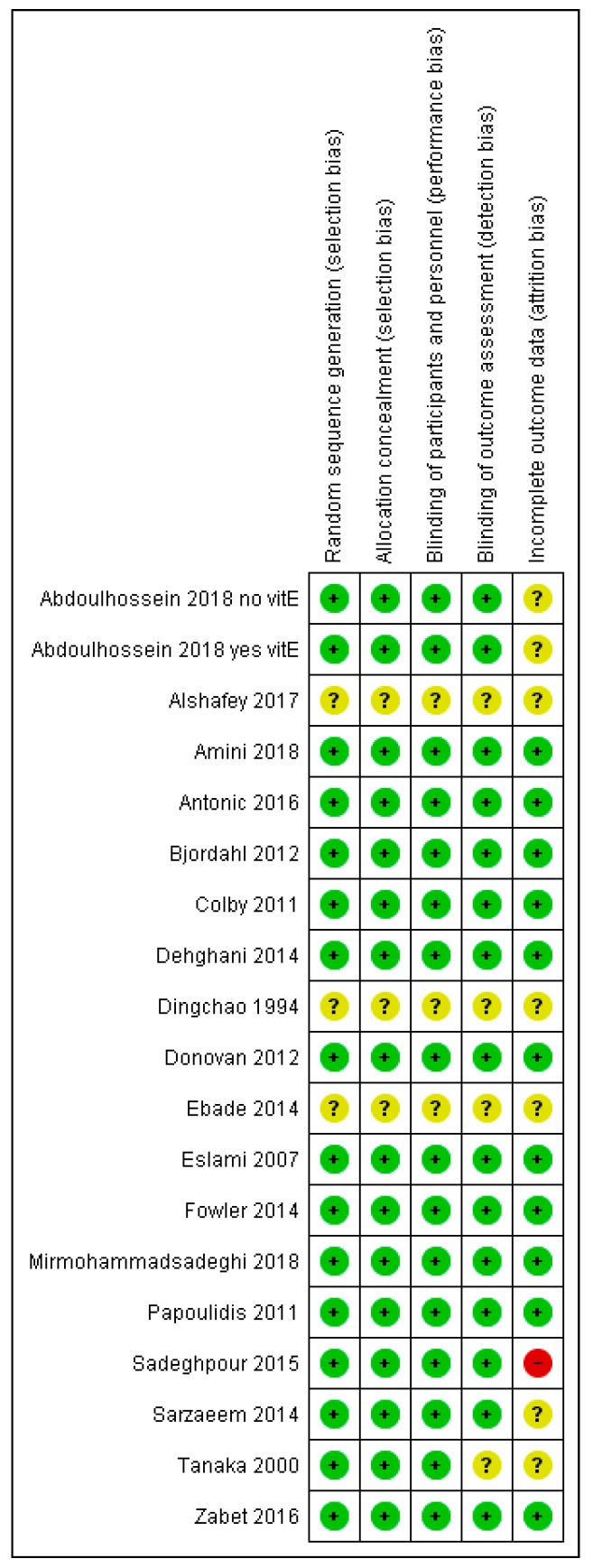
Risk of bias summary. Review authors’ judgments about each risk of bias item for each included trial. A plus mark (+) indicates that there is no substantial concern for bias in the particular quality item. A question mark (?) indicates that conclusions are unable to be drawn regarding potential bias. A minus sign (−) indicates that there is concern regarding bias. The Dingchao [106], Ebade [114], and Alshafey [118] trials were particularly poorly reported; see Table S1 in Supplementary file S1. We tried unsuccessfully to contact Dr. Alshafey and Dr. Ebade to ask for details of their methods. We did not try to contact Dr. Dingchao, since the study is old. The reference numbers to the trials are shown in Table 1.

Both authors assessed the quality of included trials for the following criteria: random sequence generation, allocation concealment, blinding of patients and personnel, blinding of outcome assessment, and incomplete outcome data (Figure 2; Table S1). Each quality item was assigned a low, unclear, or high risk of bias. Only one study had a high risk assessment on any item, and that study was excluded because of severe violation of the ITT principle [123].

### 2.5. Statistical Methods

**Figure 3:**
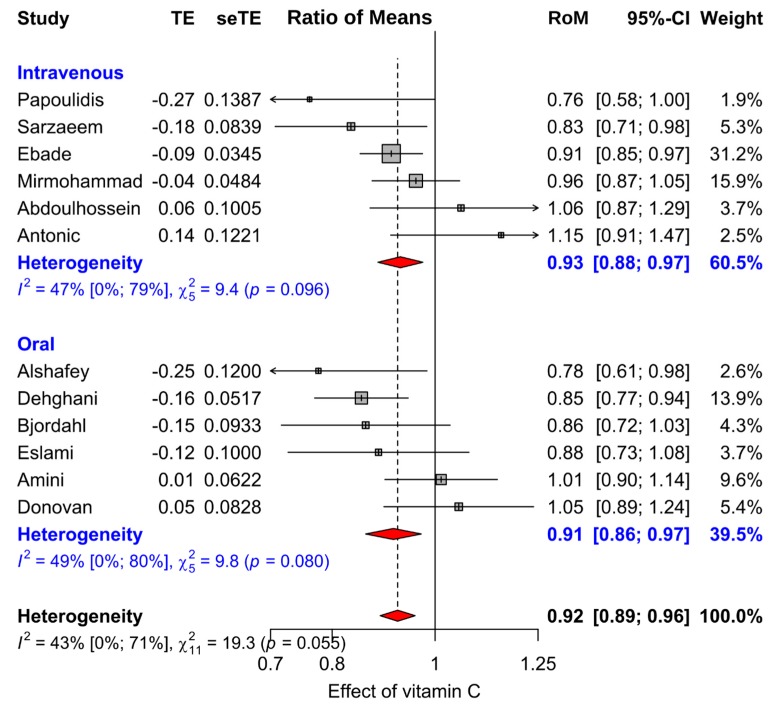
The effect of vitamin C supplementation on the length of ICU stay. This meta-analysis is termed final meta-analysis in Table 2. Subgroups of this set of 12 trials are shown in Table 2. The horizontal lines indicate the 95% CI for the vitamin C effect and the squares in the middle of the horizontal lines indicate the point estimates of the effect in the particular trial. The diamond shape indicates the pooled effect and its 95% CI. When the squares and diamonds are on the left-hand side of the vertical control level, they indicate that vitamin C is better than control. The reference numbers to the trials are shown in Table 1. Abbreviations: RoM, ratio of means; TE, logarithm of RoM; seTE, the standard error of TE; see ref. [127].

**Figure 4:**
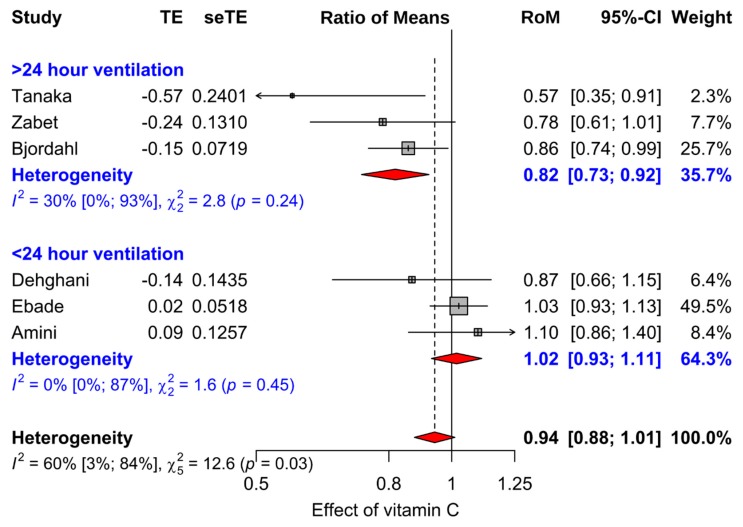
The effect of vitamin C supplementation on the length of mechanical ventilation. The two subgroups are formed by the duration of mechanical ventilation in the control group, see Table 1. The modification of vitamin C effect on the duration of mechanical ventilation was also analyzed by meta-regression over the control group duration of ventilation, and significant modification was found (*p* = 0.0013), see Supplementary file S1. The horizontal lines indicate the 95% CI for the vitamin C effect and the squares in the middle of the horizontal lines indicate the point estimate of the effect in the particular trial. The diamond shape indicates the pooled effect and its 95% CI. The reference numbers to the trials are shown in Table 1. Abbreviations: RoM, ratio of means; TE, logarithm of RoM; seTE, the standard error of TE; see ref. [127].

We analyzed the duration data on the relative scale, since there is considerable evidence that the relative scale is usually more informative than the absolute scale for the analysis of continuous outcomes [127,128,129,130]. In particular, the relative scale has been shown to be superior for the analysis of duration data [38,129,130]. We calculated the ratio of means (RoM) for the comparison of the vitamin C and control groups [127]. For example, a duration of 5 days in the control group and 4.5 days in the intervention group corresponds to RoM = 0.90 (=4.5/5). Furthermore, RoM = 0.90 corresponds to an average 10% decrease in duration. We used the Taylor series approach [127] to calculate the log(RoM)(=TE in Figure 3 and Figure 4) and the SE(log(RoM))(=seTE) (Supplementary file S2).

We pooled the selected trials with the meta procedure of the meta program package of the R statistical software [131], using the inverse variance, fixed effect options. We used the *χ*^2^ test and the *I*^2^ statistic to assess statistical heterogeneity among the trials in each meta-analysis [132]. Values of *I*^2^ greater than about 50% indicate moderate heterogeneity, and over 75% indicate a high level of heterogeneity. We used two-tailed *p* values in this analysis.

Some included studies used the Mann–Whitney (MW) test in their calculation of the *p*-values, since, for skewed data such as the length of ICU stay, it is preferable to the t-test. In some trials, the MW *p*-values were incompatible with the reported SD values. Therefore, to keep our analyses consistent with the MW *p*-values, for five studies [108,110,113,114,121], we calculated SE(log(RoM)) for length of ICU stay from the reported MW *p*-value (Supplementary file S2). For one study, we used the reported *p*-value, since that was more conservative than the *p*-value calculated from the published SD values for ICU stay [118] (see Table S1). For another study [111], we calculated SE(log(RoM)) for the duration of mechanical ventilation from the reported MW *p*-value.

In the control group of the Amini study [120], the SD was 7.36 days for the ICU length of stay, but just 0.78 to 1.5 days in three other groups. In the control group, the SD was 157.5 h for the duration of mechanical ventilation, but just 3 to 27 h for three other groups. Dr. Amini kindly sent us their data set and we found that there was one patient in the control group who had an ICU length of stay of 64 days and ventilation time of 1333 h. We removed this outlier and calculated that the mean ICU length of stay was 2.33 (SD 0.85) days and mean ventilation time was 6.68 (SD 4.26) hours in the control group. These SD values are consistent with the three other trial arms.

Abdoulhossein [122] reported a 2 × 2 factorial trial with vitamin C and vitamin E, but did not calculate the *p*-value for the interaction between the two vitamins. We were unable to contact Dr. Abdoulhossein. We used two approaches to calculate the interaction test *p*-value. First, we generated a data set with identical mean and SD values and used linear modeling to calculate the interaction *p*-value. Second, we calculated the F-value numerically from the reported means and SD values (see Supplementary files S1 and S2). They gave identical *p*-values.

## 3. Results

### 3.1. Description of the Trials

We identified 18 publications, which reported 19 trials on the effect of vitamin C on the length of ICU stay or on the duration of mechanical ventilation (Figure 1; Table 1). The trials are summarized in Table S1 of Supplementary file S1. One trial was excluded because of severe violation of the ITT principle [123]; thus, 18 trials remained for our analyses.

Length of ICU stay was studied in 17 trials, and duration of mechanical ventilation in six trials. Patients undergoing cardiac surgery were studied in 13 trials, patients with sepsis in two trials, lung contusion patients in two trials, and burns patients in one trial.

Eight trials were carried out in Iran, four in the USA, two in Egypt, and one trial in each of China, Greece, Japan, and Slovenia. The total number of patients in the 18 trials was 2004, with 1835 patients in studies related to cardiac surgery and 169 patients in other settings. The mean age in the trials ranged from 40 to 70 years (median 60 years). Of the trials that reported sex distribution, the proportion of males varied from 57% to 79% (median 70%) (Table S1).

Vitamin C was administered orally in seven trials and intravenously in 11 trials. All oral administration trials investigated cardiac patients and the dose ranged from 1 to 3 g/day. The 11 intravenous administration trials include all five non-cardiac studies and the dose ranged from 0.5 to 110 g/day. Three trials administered vitamin C on one single day only, and 11 administered vitamin C for four days or longer (Table 1 and Table S1).

There was substantial variation in patient selection between the trials (Table 1). The cardiac trials enrolled mostly patients undergoing elective surgery and so the patients were in reasonably good health and the length of ICU stay was short; one trial enrolled both elective and urgent cardiac surgery patients [112]. In nine cardiac surgery trials the duration of ICU stay was two days or less in the control group, whereas in four trials the stay was three–four days. In the two lung contusion trials, the mean ICU stay was five days in the control groups. The longest reported ICU stay was in the two trials that enrolled patients with sepsis, with ICU stay of 11 days [115] and 20 days [117]. The trial with burns patients did not report the duration of ICU stay, but the duration of mechanical ventilation was 21 days in the control group [107], which is about 10 times longer than the ICU stay in the majority of cardiac surgery trials. Thus, ICU stay was evidently the longest in the trial with the burns patients [107].

The majority of the trials were randomized (Table S1). Two trials used quasi-randomization. Tanaka et al. allocated patients to study groups by the month of admission [107] and Mirmohammadsadeghi allocated patients by their person numbers [121]. The method of allocation was not reported by Ebade et al. [114] and by Alsfahey [118]. Most trials reported no or few drop-outs (Table S1).

The risk of bias assessment of the trials is shown in Figure 2. The justifications for the quality assessments are shown in Table S1. An explicit placebo was used in nine of the trials [107,109,110,111,112,114,115,116,117]. Nevertheless, ICU patients receive a large number of treatments, and thus, it is highly unlikely that receiving one additional tablet or infusion would produce a placebo effect that could influence the length of ICU stay. Therefore, we did not consider that the lack of a placebo undermines the validity of the comparisons in those trials that did not have placebo. In 14 trials we concluded that there was no reason to assume that blinding at the stage of allocation, in the treatment phase, or in the outcome assessment might have led to bias in the findings.

Four trials did not report the methods in sufficient detail to enable conclusions to be drawn about the possibility of bias caused by poor blinding [106,107,114,118]. In our sensitivity analyses for vitamin C effect on ICU stay, we excluded the two particularly poorly reported trials [114,118], and a quasi-randomized trial [121].

Although plasma vitamin C levels at baseline and during treatment are highly informative characteristics of studies on vitamin C, only one trial reported plasma levels at the baseline. Fowler reported that at enrollment the mean plasma vitamin C level of their patients was 18 μmol/L [115]. Plasma vitamin C level in the placebo group fell from 20.2 μmol/L at entry to 15.6 μmol/L on study day four. In contrast, on day four, the plasma vitamin C in the 3.5 g/day group had increased to 330 μmol/L, and in the 14 g/day group to 3080 μmol/L [115]. Tanaka published plasma vitamin C levels during the study in a figure but did not provide numbers. With the intravenous administration of 66 mg/hour/kg over a 24 h treatment period, the plasma vitamin C level increased to about 500 μmol/L in the vitamin C group, but remained close to zero in the placebo group [107].

### 3.2. Effect of Vitamin C Administration on the Length of ICU Stay

In our meta-analyses we pooled the results of the trials on the relative scale, calculating the ratio of means (RoM). For small effects, it is useful to transform the RoM values to a percentage scale. For example, RoM = 0.922 corresponds to a 7.8% mean decrease in the length of ICU stay.

Over the 17 trials reporting on length of ICU stay, there is highly significant heterogeneity in the effect of vitamin C with *I*^2^ = 90% (*p* = 10^−24^) (Table 2 and Figure S1 in Supplementary file S1). The singular reason for this very high heterogeneity is the oldest trial by Dingchao (1994) that was carried out in China [106]. When it was excluded, the level of heterogeneity decreased dramatically to *I*^2^ = 58% (*p* = 0.002). In addition, when the Abdoulhossein (2018) trial with patients administered vitamin E [122] was also excluded, the level of heterogeneity decreased further to *I*^2^ = 35% (*p* = 0.088). Finally, three trials were very small [109,115,117] (Table 1), with a total of just 76 patients; with each of them having weight <1% in the meta-analysis that included all trials in Figure S1. These trials did not contribute to the estimate of effect, but they inflated the degrees of freedom for the test of heterogeneity (Table 2). Thus, their exclusion had no influence on the point estimate or the *p*-value, but moderately increased heterogeneity. We also excluded these three trials, and our final meta-analysis is shown in Figure 3. The excluded five trials are discussed separately below and the forest plot of all 17 trials is shown in Figure S1.

In the final meta-analysis of 12 trials in Figure 3, vitamin C shortened the duration of ICU stay on average by 7.8% (95% CI: 4.2% to 11.2%; *p* = 0.00003). There are substantial variations in the trials of the final meta-analysis, and moderate statistical heterogeneity over the 12 studies with *I*^2^ = 43%. Nevertheless, the confidence intervals of all the 12 trials are consistent with the pooled effect.

We calculated the estimate of effect for selected subgroups (Table 2). In a sensitivity analysis, we excluded three trials; however, the estimate of vitamin C effect on ICU stay was essentially the same in the nine better-quality trials compared with the final meta-analysis of the 12 trials: 7.3% versus 7.8%, respectively. Eleven of the 12 trials investigated ICU stay associated with cardiac surgery. Six trials administered vitamin C orally and six administered intravenously (Figure 3). Six of the trials were carried out in Iran, and six outside of Iran. For each of these subgroup selections, the estimate of effect ranged from a statistically significant 7% to 8% reduction in the length of ICU stay.

All of the six oral administration trials used vitamin C doses of 1 to 3 g/day. Overall, in these low dose oral administration studies, vitamin C reduced the length of ICU stay by 8.6% (95% CI: 3.0% to 14.0%; *p* = 0.003). For these six trials, the weighted mean dose of vitamin C was 2.0 g/day. Thus, the effective dose of 2.0 g/day was associated with the 8.6% reduction in ICU stay.

In seven trials with control group ICU stay from 1 to 2 days, corresponding to less sick patients, vitamin C reduced ICU stay by 5.7% (*p* = 0.027). In five trials with control group ICU stay from 3 to 5 days, corresponding to sicker patients, vitamin C reduced ICU stay by 10.1% (*p* = 0.0001). However, the confidence intervals for these groups were substantially overlapping (Table 2), and the subgroup comparison gives *p* = 0.21 (see Supplementary file S1). We did not carry out meta-regression by the dose of vitamin C, since few trials used doses over 3 g/day (Table 1). In addition, we did not carry out meta-regression by the proportion of males, since the range was very narrow in the studies (57% to 79% males) (Table S1).

Five of the 17 trials that reported length of ICU stay were excluded from the meta-analysis in Figure 3. The Dingchao trial is the oldest, carried out in China in the early 1990s [106]. They reported that intravenous administration of 17 g/day of vitamin C for one single day reduced the length of ICU stay by 44% (95% CI 40% to 49%). On the basis of the comparison of the confidence intervals, and the heterogeneity test, the Dingchao trial is fundamentally inconsistent with all the trials shown in Figure 3. The methods of the Dingchao trial are poorly reported; however, we do not consider that there is reasonable justification to exclude it. However, even if the study findings were valid in the context of China in the 1990s, the results cannot be generalized to the contexts examined in the more recent trials. All the more recent trials found the effect of vitamin C to be much smaller (Figure 3). Nevertheless, the Dingchao study supports the concept that vitamin C can influence ICU stay.

Abdoulhossein [122] reported a 2 × 2 factorial trial with vitamin C and vitamin E. There was a statistically significant interaction between the two vitamins (Table 3). Intravenous 0.5 g/day vitamin C shortened the duration of ICU stay only in patients who were simultaneously administered 1 g/day vitamin E. In Figure 3, we excluded the vitamin E patients of that study, because this considerably decreased the heterogeneity of the final meta-analysis (Table 2). Nevertheless, the benefit of vitamin C in the patients administered vitamin E supports the concept that vitamin C can influence ICU stay in some contexts.

Three particularly small trials [109,115,117] were excluded from Figure 3 since they had weight <1% in the meta-analysis of the 17 comparisons. They had such wide 95% CIs that they were uninformative about any possible effects of vitamin C on the length of ICU stay, see Figure S1.

### 3.3. Effect of Vitamin C on Mechanical Ventilation

We identified six trials that reported the duration of mechanical ventilation in the vitamin C and control groups (Figure 4). On average, the duration of mechanical ventilation was non-significantly shorter in vitamin C groups by 5.7% (95% CI −1.1% to 12.4%; *p* = 0.1). However, there was a significant difference in the effect of vitamin C on the duration of mechanical ventilation between those who were ventilated for less than 24 h and those who were ventilated for more than 24 h (*p* = 0.004). In meta-regression, the evidence for modification of the vitamin C effect by duration of mechanical ventilation was even stronger (*p* = 0.0013; Supplementary file S1). In three trials in which patients needed mechanical ventilation for over 24 h, vitamin C shortened the duration of mechanical ventilation by 18.2% (95% CI 7.7% to 27%; *p* = 0.001) (Figure 4). In three trials in which participants needed mechanical ventilation for less than 24 h, there was no evidence of an effect from vitamin C (*p* = 0.7). In two trials with non-cardiac patients [107,117], vitamin C shortened the duration of mechanical ventilation by 27% (95% CI 9% to 42%; *p* = 0.006).

### 3.4. Other Effects in the Included Trials

Our primary and secondary outcomes were the length of ICU stay and the duration of mechanical ventilation. Additionally, we collected data on other outcomes relevant in the ICU context; however, there are few and they are so heterogeneous that further meta-analyses were not feasible.

Two small trials with sepsis patients were not informative about ICU stay and were excluded from Figure 3 [115,117], though another of the sepsis trials contributed to the analysis of mechanical ventilation (Figure 4). Nevertheless, certain of their other outcomes are relevant for the ICU context.

Fowler [115] reported that 3.5 and 14 g/day of vitamin C for 4 days significantly reduced multi-organ failure scores, and the rate of decline with the higher dose was twice the rate with the lower dose. They also reported decreased inflammation markers C-reactive protein and procalcitonin, and the effects of the higher dose appeared greater also on these outcomes.

In another trial with sepsis patients, Zabet [117] reported that 7 g/day of vitamin C for 3 days significantly reduced the mean daily dose and mean duration of norepinephrine administration (*p* < 0.01 for both). Mortality over 28 days in the vitamin C group was reduced by RR = 0.22 (95% CI 0.06–0.85; *p* = 0.018 Fisher exact test) based on 2/14 deaths in the vitamin C group and 9/14 in the placebo group.

In the earliest study, Dingchao [106] reported that 17 g vitamin C on a single day decreased CK-MB levels and cardiac index during follow-up.

Tanaka [107] reported that in their trial with patients with severe burns, the requirement for fluid infusions in the vitamin C group was decreased by 45% (*p* < 0.01). In addition, fluid retention was decreased by 68% (*p* < 0.01) and weight gain was 48% lower (*p* < 0.01) in the vitamin C group.

Abdoulhossein [122] reported that the combination of vitamin C and E increased oxygen saturation and decreased carbon dioxide levels.

## 4. Discussion

### 4.1. The Complex Biochemistry of Vitamin C

Poor healing of wounds and swelling of the gums are the most well-known symptoms of scurvy. At the biochemical level, they are explained by the role of vitamin C in collagen metabolism. However, vitamin C has a number of biochemical roles unrelated to collagen metabolism. For example, it participates in the synthesis of norepinephrine, which is a neurotransmitter, carnitine, which is involved in energy metabolism, and nitric oxide (NO) [133,134,135,136,137,138,139]. Vitamin C participates in the terminal amidation of several neuropeptides [140,141]. There are over 100 known neuropeptides and over half of them require amidation to achieve full biological activity, such as vasopressin, oxytocin, gastrin, calcitonin, neuropeptide Y, and substance P.

Vitamin C also participates in the demethylation of DNA and histones and thereby influences the epigenome [142,143,144,145,146,147]. It has been estimated that vitamin C may demethylate over 1000 genes in embryonic stem cells [145]. Vitamin C hydroxylates specific proline residues in hypoxia-inducible factor-I, which is a transcription factor that is a key to oxygen sensing, and may regulate several hundred genes [143,147,148,149]. In addition, vitamin C is a major water-soluble antioxidant, and thereby, can have a wide range of nonspecific effects. Such biochemical effects translate to vitamin C participating in higher-level physiological processes, for example, in the immune system [142,147,150,151], endothelial functions [18,19,20,143,152,153], the nervous system including pain sensation [44,45,46,47,136,139,154,155], and in the functioning of the heart [22,23,24,25,26,27,28,29,156,157,158,159]. These are unrelated to collagen metabolism.

The numerous biochemical effects of vitamin C can explain the large variety of symptoms that have been reported in experimental vitamin C deficiency studies, such as depression, anxiety, irritability, confusion, shortness of breath, fatigue, oliguria with edema especially of the lower extremities, anemia, bruising, hemorrhages, joint pains, joint effusions, peripheral neuropathy, and common colds that last longer than usual [50,51,52,53,54,55,56,57,58,59,60,61,62].

According to case reports of scurvy, in addition to fatigue, anemia, bruising, hematomas, and different types of pain, vitamin C deficiency has been associated with various cardiovascular symptoms [67,68,69,70,71,72,73,74,75,76], dyspnea [64,70,71,72,73,74,75,76,77,78,79,80,81], lower limb edema [13,64,66,70,72,80,160,161,162,163,164,165,166,167,168,169,170,171,172,173,174,175], infection-type symptoms [66,67,68,73,160,161,165,167,174,175,176,177], bleeding in the gastrointestinal and other regions [76,164,172,175,177,178,179,180,181], and neuropsychiatric symptoms [70,71,182,183,184,185].

Logically, low systemic vitamin C levels should be considered in the differential diagnosis of these symptoms observed in experimental vitamin C deficiency studies and in case reports of scurvy. Scurvy is a clinical diagnosis and not a diagnosis based on plasma vitamin C level. For example, Hodges concluded that “the subjects … had plasma values well above the deficiency level throughout the time they had frank scurvy. Furthermore, a comparison between plasma levels of ascorbate and pool sizes showed a very poor correlation… In general, it is fair to say that scurvy appeared when the body pool fell below 300 mg” ([56], p. 441). The most undisputable evidence of scurvy symptoms is that they disappear with vitamin C administration [13,64,65,66,67,68,69,70,71,72,73,74,75,76,77,78,79,80,81,160,161,162,163,164,165,166,167,168,169,170,171,172,173,174,175,176,177,178,179,180,181,182,183,184,185].

### 4.2. Early Evidence of Vitamin C Deficiency in Hospital Patients

In the 1930s, after vitamin C had been identified, there were reports stating that plasma vitamin C levels of hospital patients were much lower than those of healthy people [186,187,188]. Finkle (1937) stated that “a large percentage of the population encountered in the wards and outpatient department of the Hospital were found to have excretion levels of vitamin C in the urine considerably below that found in normal subjects living on an adequate mixed diet. When such individuals receive 100 mg of vitamin C intravenously, there is practically no rise in urinary excretion of vitamin C” [186] (p. 593). Decrease in vitamin C levels after surgery was reported by Lund (1939) who wrote that “plasma cevitamic acid determinations were made before and after operation on 43 patients coming to major operation. In nearly every case there was a prompt fall after operation from the original level” [187] (p. 127). Abbasy et al. (1937) wrote that “in common with other infectious conditions, osteomyelitis causes a diminished rate of excretion of vitamin C in the urine and a lowered response to test dose, indicative of an apparently increased usage of the vitamin during the infective process” [188] (p. 180). In addition, Evans (1938) reported that vitamin C increased the urinary output of patients with heart failure [189,190].

At that time, the concept of oxidative stress was unknown, as were many biochemical and physiological functions of vitamin C that were identified much later. Thus, the biochemical concepts required to consider the potential physiological harms of very low vitamin C levels were not developed. Randomized trial methodology had also not evolved, to enable an unbiased scientific investigation of whether vitamin C administration actually benefits hospital patients with very low vitamin C levels. A low vitamin C level does not necessarily mean that administration of vitamin C would be beneficial. A particular vitamin C level may simply be a marker for the various pathological processes rather than being implicated in the actual processes themselves. If this was the case, there would be no benefit from administration of vitamin C. Nevertheless, low levels of vitamin C in hospital patients still occur [82,83,84,85,86,87,88,89], and there is modern evidence that vitamin C metabolism is changed under various forms of severe physiological stress such as surgery, sepsis, and trauma [90,91,92,93,94,95,96,97,98,99,100,101,102,103,104,105]. Hence, the question of the clinical importance of low vitamin C levels in hospital patients is as relevant today as it was in the 1930s.

### 4.3. Vitamin C and Pneumonia and the Common Cold

Before vitamin C was identified, scurvy was associated with an elevated risk of pneumonia [2,48], which suggests that vitamin C might have clinically relevant effects on infections. After the isolation of vitamin C, a few German and US physicians proposed that it might be beneficial in the treatment of pneumonia [191,192,193,194,195,196,197]. However, to our knowledge, only two small controlled trials published decades ago have investigated the effect of vitamin C for treating pneumonia.

A Russian study reported a linear dose response over two vitamin C doses on the length of hospital stay for pneumonia patients [49,198]. A Scottish study reported significant benefit on the respiratory symptoms of pneumonia patients, with only one death in the vitamin C group compared with five in the placebo group [49,82]. A further study found that vitamin C reduced the incidence of pneumonia by 80% in influenza A patients [49,199]. Although these studies were small and had methodological deficiencies, they provided significant justification for the need for further research into the effects of vitamin C on pneumonia [200]. While this was over a decade ago, no new controlled trials on vitamin C and pneumonia have been published since.

The leap from the common cold to critical care is vast; however, the ubiquity and mild character of the common cold has led to a large number of studies of vitamin C for prevention and treatment being undertaken. These studies include a total of more than 10,000 participants and such a large data set allows for informative subgroup analyses and other comparisons [16,41,42]. There are some lessons from the common cold studies that may be informative in the ICU context.

Firstly, the common cold leads to a decline in vitamin C levels in white blood cells, and while 0.2 g/day of vitamin C did not return the levels to normal, administration of 6 g/day did [201]. Thus, it seems that even mild infections can increase vitamin C metabolism such that systemic vitamin C levels are reduced.

Secondly, there is evidence that the effect of vitamin C supplementation on common cold incidence is context dependent. Context-dependency means that we should not expect a uniform effect in all populations. Although vitamin C has no effect on the incidence of colds in the normal population, it halved the incidence of colds in participants undergoing heavy physical activity [41,42,43,202], and it decreased the incidence of colds in males who had particularly low dietary vitamin C intake [16,202,203]. Vitamin C administration has shortened the duration of colds which indicates that taking more vitamin C is beneficial when there is an infection ongoing [36,37,38,39,40,41,42,204]. Heavy physical activity and infections such as the common cold lead to oxidative stress, which may explain the benefits of vitamin C administration in these contexts. Furthermore, vitamin C supplementation may be beneficial for those who have particularly low intake levels.

Thirdly, two common cold studies compared two different vitamin C doses, and both found linear dose-dependency [40,42,204]: 6 g/day was twice as effective as 3 g/day in shortening the common cold [205], and administration of 8 g on a single day was twice as effective as 4 g [206]. This indicates that even for a mild infection such as the common cold, doses of 3–4 g per day of vitamin C may not be sufficient for maximum benefit. The common cold induces only low-level physiological stress, and so it is possible that more severe medical conditions may benefit from higher doses than 6–8 g/day of vitamin C. As noted above, dose-dependency was also reported in an old pneumonia study [49,198].

Fourthly, some common cold studies have indicated that the effects of vitamin C may be greater for outcomes that reflect greater severity [204]. In two large studies, vitamin C did not shorten the duration of runny nose, but significantly decreased the days off work in adults [207] and the days off school in children [208].

Finally, a few common cold studies have indicated that in some contexts vitamin C administration may lead to greater effects in males than in females [202,209,210,211,212].

### 4.4. Vitamin C and the Cardiovascular System

A meta-analysis found that vitamin C reduced post-operative atrial fibrillation (POAF) in trials conducted outside of the USA, but not in studies carried out in the USA, indicating context dependency of the effect [28]. For several treatments, substantial divergence in the size of effect has been found between more developed and less developed countries [213], and that may be the case also for vitamin C and POAF.

In patients undergoing cardiac surgery, vitamin C has increased cardiac perfusion after the operation [23] and decreased the level of markers of myocardial injury, such as CK-MB (creatine kinase MB isoenzyme) [22,106,214]. A meta-analysis of patients with atherosclerosis or heart failure found that vitamin C improved endothelial function [18]. In a few trials, vitamin C increased the left ventricular ejection fraction [22,23,24,25,26]. A meta-analysis showed that vitamin C supplementation can lower blood pressure [21]. Vitamin C may also protect against myocardial ischemia/reperfusion injury [215].

In one of the experimental scurvy studies, two participants developed acute cardiac complications, and a third developed shortness of breath and pain in the chest. Therefore, the trial was terminated for each of them, and all three showed striking improvements within a few days of receiving vitamin C [53]. Several case reports have indicated that vitamin C deficiency can cause cardiovascular effects such as hypotension, hypertension, right heart failure, and ECG changes [67,68,69,70,71,72,73,74,75,76].

### 4.5. Other Possible Effects of Vitamin C Relevant in the ICU Context

Pneumonia is the most common cause for severe sepsis, accounting for about half of all cases [216]. As described above, vitamin C may be effective in the prevention and treatment of pneumonia in some contexts.

Vitamin C has been beneficial in animal models of sepsis, for example, by decreasing mortality [152,153,217]. In one study, intravenous infusion of vitamin C increased the free radical form of vitamin C significantly more in sepsis patients than in healthy subjects, indicating markedly different handling of vitamin C in the two groups [94]. Recent reviews have discussed the rationale for investigating the effects of vitamin C as a treatment for sepsis [140,218,219,220,221,222,223].

The highest vitamin C levels in the body are in the adrenal glands and brain [154]. Adrenal glands produce hormones that participate in stress responses. Vitamin C deficiency can lead to symptoms reflecting the effects of vitamin C in the nervous system [61,70,71,155,182,183,184,185]. In two controlled trials, vitamin C administration improved the mood of patients hospitalized with acute illnesses [224,225]. Thus, in addition to somatic effects, vitamin C can have psychological effects that could be beneficial for critically ill patients and might also influence their duration of ICU stay.

### 4.6. Pharmacokinetics of Vitamin C

Oral and intravenous administration of vitamin C lead to substantially different plasma vitamin C levels. When 1.25 g of vitamin C was given intravenously, plasma peak level increased approximately six-fold compared with the level after oral administration of the same dose [226,227]. Because of this, intravenous vitamin C has been used and investigated by some physicians [106,107,110,114,116,117,119,122,192,193,194,195,196,228,229,230,231,232,233,234,235]. Thus, the two methods of administration may lead to different clinical effects even if the dose is the same. Paradoxically, a previous meta-analysis of vitamin C on POAF and hospital stay found that the effects of the two routes were divergent [28]. Oral vitamin C appeared to cause a greater decrease in the incidence of POAF, whereas intravenous vitamin C led to a greater decrease in hospital stay.

### 4.7. Current Findings: Vitamin C and Length of ICU Stay and Duration of Mechanical Ventilation

There are a variety of biochemical reactions in which vitamin C plays a part, a large number of physiological processes that are consequently influenced, and a large number of clinical effects of vitamin C that have been observed in controlled trials [18,19,20,21,22,23,24,25,26,27,28,29,30,31,32,33,34,35,36,37,38,39,40,41,42,43,44,45,46,47,48,49]. Given this, and the evidence for low vitamin C levels and increased metabolism of vitamin C in critically ill patients [90,91,92,93,94,95,96,97,98,99,100,101,102,103,104,105], it seemed reasonable to examine whether vitamin C influences practical outcomes, such as the length of ICU stay and the duration of mechanical ventilation without any restrictions on specific medical conditions.

In our meta-analysis of 12 controlled trials with 1766 patients, we found that vitamin C shortened the duration of ICU stay on average by 8% (Figure 3). Meta-analysis of six trials that reported the duration of mechanical ventilation found that vitamin C shortened the duration by 8% (Figure 4).

We did not restrict our study to randomized and placebo-controlled trials. Although methodological details should be reported so that readers can draw their own conclusions about the validity of studies [236], there is empirical evidence that often trials are better than the publications indicate [237,238,239]. Therefore, we decided to include studies without strict methodological requirements, and instead used sensitivity analysis to test whether the exclusion of the methodologically less satisfactory studies resulted in a decreased estimate of effect.

In empirical analyses, the placebo-effect has not been quantitatively meaningful when the outcomes were objective [240]. In particular, in an ICU context, the patients are given a number of treatments and it is unlikely that getting one more tablet or infusion would generate a quantitatively relevant placebo effect. In addition, usually ICU patients are so unwell that they are quite often unaware of what treatment they are receiving.

Most of the included trials were randomized (Figure 2, Table S1). Two studies [114,118] did not report the method of allocation. The trial arms had an identical size, and thus, it is possible that alternative allocation was used. Tanaka allocated patients to groups by the month [107]; however, it does not seem reasonable to assume that such an allocation necessarily led to systematic bias between study groups, when the study lasted for years. Mirmohammadsadeghi [121] allocated patients to groups by the personal number of the patients. The purpose of random allocation is to generate study groups that are balanced, and the reported baseline data were balanced in each of the four trials. In any case, in our sensitivity analysis on ICU stay, we excluded three of these studies [114,118,121]; however, there was no material difference in the point estimates of effect (Table 2). The fourth study did not report duration of ICU stay [107].

The calculation of the 8% average decrease in ICU stay is reasonable when testing the null hypothesis that vitamin C has no effect on ICU stay. However, that estimate cannot be generalized to all ICU contexts, since the patients treated in ICU are highly diverse. All except one of the 12 trials in our main meta-analysis on ICU stay were with patients undergoing elective cardiac surgery (Figure 3). However, two non-cardiac trials found a 27% decrease in the duration of mechanical ventilation (Figure 4), indicating that the effects of vitamin C are not limited to cardiac patients. Furthermore, two trials with sepsis patients found other benefits from vitamin C [115,117]. In addition, a trial with lung contusion patients found that vitamin C was beneficial when patients were also given vitamin E (Table 3).

We did not find any meaningful differences between the effects of oral and intravenous vitamin C (Table 2); however, the confidence intervals are wide, and no firm conclusions should be drawn. Six oral administration trials found an 8% reduction in ICU stay with a mean dose of 2 g/day. The data was too limited to carry out meta-regression by vitamin C dosage; however, the possibility of greater benefits with higher doses should be considered, since linear dose-dependency for 6 to 8 g/day has been found in two common cold studies [40,42,204,205,206]. Common cold studies have indicated differences between males and females in some contexts [202,209,210,211,212]; however, the range of variation in the proportion of males in the studies included in this meta-analysis is so small that a meta-regression was not meaningful.

The effect of vitamin C administration on the duration of mechanical ventilation was significantly greater for ICU patients who were more severely ill (Figure 4). We also found a greater effect of vitamin C in trials in which the length of ICU stay was longer, corresponding to sicker patients, compared with the effect in trials in which the length of ICU stay was shorter (Table 2). This is also consistent with vitamin C having greater effect on more severely ill patients.

Given that all people obtain vitamin C from their food, and there is great variation in vitamin C intake, it is surprising that only one of the included trials measured and reported baseline vitamin C levels. Therefore, the possible role of baseline vitamin C level as a variable defining the benefit of giving vitamin C could not be investigated.

### 4.8. Other Relevant Studies

There are studies not included in our systematic review that are nevertheless informative on this topic. Two controlled trials with critical care patients did not report the length of ICU stay or duration of mechanical ventilation but reported other benefits from vitamin C [241,242].

A controlled trial carried out in Bangladesh reported that 1 g/day intravenous vitamin C decreased mortality for tetanus by 100% in 62 patients aged 1 to 12 years, and by 45% in 55 patients aged 13 years and more [241]. The greater benefit for younger children is consistent with dose dependency. Young children weigh less, and therefore, 1 g/day corresponds to a greater dose per weight. Although the study had various shortcomings, the benefits are not easily explained by the methodological deficiencies [243]. Furthermore, the concept that vitamin C may influence tetanus is supported by animal studies in which vitamin C has protected against tetanus toxin and other clostridial toxins [244,245].

A randomized trial of 84 patients with acute pancreatitis compared 1 g/day and 10 g/day intravenous vitamin C for five days [242]. The higher dose significantly shortened the duration of fever by 26%, vomiting by 16%, and the length of hospital stay by 30%. Given that the control group was also given quite a high dose of vitamin C, this study indicates dose dependency even at higher doses.

In addition to trials with vitamin C alone, there are controlled trials of vitamin C given with other antioxidants. However, if there is a benefit from a set of several antioxidants, it is not possible to tell which one of them was effective, or whether an interaction between several antioxidants led to the benefit as in Table 3. Similarly, if there is no benefit in another study with several different antioxidants, we do not know whether some of the components alone might be beneficial; another component may have prevented the benefit. The latter is not pure speculation as vitamin E supplementation was harmful for middle aged males with high dietary vitamin C intake, suggesting harmful interaction in certain contexts [246]. Nevertheless, certain studies have reported that mixtures containing vitamin C were beneficial for critically ill patients [247,248,249,250,251,252,253].

Furthermore, some before-after studies have reported benefits from vitamin C or from vitamin C together with other antioxidants. However, there can be various temporal changes and other confounders that could bias before-after studies. The concern regarding bias in observational studies is the reason that evidence-based medicine focuses on controlled trials. However, if the differences in a before-after comparison are very large, it is not easy to explain them by confounding. Some before-after studies were consistent with the notion that vitamin C may be beneficial for critically ill patients [254,255,256,257,258,259,260]. In one study with 4294 acutely injured patients [254], the correct number-needed-to-treat to prevent one death with vitamins C and E and selenium was 17 and not 1710 as reported by the authors [261].

### 4.9. Safety of Vitamin C

In the US nutritional recommendations, the ’tolerable upper intake level’ of adults for vitamin C was stated to be 2 g/day. However, the basis for this upper limit is the appearance of diarrhea [63] which is a trivial adverse effect. Furthermore, consistent with changes in vitamin C metabolism, patients with various diseases can take much higher doses before they get diarrhea [262].

Several studies have shown that 4 g/day of vitamin C increases plasma levels of critically ill patients to the levels of the general population [102,103,104,105]. Furthermore, all the studies in Figure 3 used doses of 3 g/day or less. Hence, there should not be any safety concerns if the goal of treatment is to reverse the particularly low vitamin C levels in ICU patients.

High doses of vitamin C, over 10 g/day, and in some cases even over 100 g/day, have been administered both orally and intravenously for patients with low frequency of adverse effects [228,229,230,231,232,233,234,235,262]. However, there are cases of oxalate nephropathy caused by very high doses of vitamin C. In two cases, oxalate nephropathy appeared as the cause of death; however, one of the patients was administered 101 g of vitamin C intravenously in 18 h, and the other was administered 224 g in 20 h [263]. The patients had burns to more than 60% of their bodies and it is not clear what role the vitamin C played in their deaths versus the very severe burns. In any case, it seems evident that doses substantially higher than 3 g/day should be administered with caution, e.g., by monitoring serum oxalate levels.

The rare cases of oxalate nephropathy caused by particularly high doses of vitamin C should not be used as a counterargument for raising very low vitamin C plasma levels of ICU patients to the range of normal healthy people with doses of about 4 g/day [102,103,104,105].

### 4.10. Preconceptions about Vitamin C: Why Is Vitamin C not Used Despite the Evidence?

In 1975, Thomas Chalmers published a meta-analysis in which he pooled the findings of eight vitamin C and common cold trials and calculated that colds were 0.11 (SE 0.24) days shorter in the vitamin C groups [264]. Chalmers concluded from the small effect and the large standard error that there was no evidence that vitamin C was effective for reducing the duration of the common cold. That review was highly influential and has been cited in textbooks on nutrition and infectious diseases, and in nutritional recommendations [38,265]. For example, in 1987, the American Medical Association based its official statement that “One of the most widely misused vitamins is ascorbic acid. There is no reliable evidence that large doses of ascorbic acid prevent colds or shorten their duration” wholly on Chalmers’ review [266] (p. 1934).

However, in 1995 the Chalmers review was shown to be seriously flawed. There were errors in the extraction of data, inconsistency in the selection of studies for the meta-analysis, and errors in the calculations [38,265]. If the Chalmers review had included only studies in which the vitamin C dose was 1 g/day or greater, had extracted data correctly, and calculated the results correctly, then the estimate of effect would have been substantially higher: the effect of vitamin C would have been 0.93 (SE 0.22; *p* = 0.01) days reduction in the duration of colds [38]. Unfortunately, the textbook authors, authors of the nutritional recommendations, and experts of the American Medical Association at that time did not look critically at the original scientific literature on vitamin C and the common cold, and thus missed the errors in Chalmers’ review.

Another influential and frequently cited review by Dykes and Meier from 1975 on vitamin C and the common cold [267] was also flawed [39,265]. Linus Pauling also pointed out several problems in the Dykes and Meier review and submitted a manuscript to the Journal of the American Medical Association (JAMA); however, his paper was rejected even after Pauling twice made revisions to meet the suggestions of the referees. The manuscript was finally published in a minor journal [268,269]. The rejection of Pauling’s review by JAMA was disappointing, since the readers were thereby prevented from seeing the other side of an important scientific controversy. In addition, a highly influential randomized trial on vitamin C and the common cold, also from 1975 [205] was analyzed erroneously [40,265,270,271].

The problems with the two reviews and with the analysis of the randomized trial may be partly explained by the preconceptions of the authors. Furthermore, the very wide acceptance of the two reviews and the randomized trial in mainstream medicine, e.g., reflected by their usage in textbooks and nutritional recommendations etc. [265], suggests that the readers probably also had strong preconceptions against vitamin C being beneficial; otherwise they might have read the texts with a more critical eye.

Prejudice against vitamin C is not limited to the common cold. Richards documented bias against vitamin C when she compared the attitudes and arguments of physicians to three putative cancer medicines: 5-fluorouracil, interferon, and vitamin C [272,273,274].

In an analysis discussing the preconceptions in academic medicine on micronutrient supplements, Goodwin and Tangum gave several examples to support the conclusion that there has been systematic bias against the concept that vitamins might be beneficial in levels higher than the minimum required to avoid classic deficiency diseases [275]. In other papers, Goodwin and Goodwin reviewed several cases in which an effective method of treatment was erroneously rejected: the rejection seemed to be caused by the lack of understanding of the physiological mechanism of the effect [276,277]. This may also apply to the prejudices against vitamin C being beneficial in higher doses for diseases other than scurvy. The effects of vitamin C on scurvy were explainable by its effects on collagen metabolism. However, with the early understanding of biochemistry it was not evident how vitamin C could have an effect on infections, cancer, or other diseases that did not involve collagen metabolism. Thus, it seems that the limited biochemical understanding may have been one reason why the early findings [2,186,187,188,189,190,191,192,193,194,195,196,197,198,199,244] were not pursued.

Using vitamin C to prevent or treat diseases is often classified in the field of alternative medicine. However, such a classification does not mean that vitamin C is ineffective for all conditions other than scurvy [278].

## 5. Conclusions: The Way Forward

We found statistically highly significant evidence that vitamin C can shorten the length of ICU stay. We consider that our finding is a proof of concept, strongly encouraging further research, rather than justifying recommendations for change in practice. In further studies, the dose-response relationship should be carefully investigated, and oral and intravenous administration should be compared directly. Given that some common cold studies found the benefit of vitamin C to be greater in males than females, the effects of vitamin C in the ICU context should be compared between sexes. ICU patients are a highly heterogeneous group, and evidently, one estimate of vitamin C effect should not be expected to apply to all patient groups. Our analysis on mechanical ventilation indicated that the benefits of vitamin C may be greater for patients with more severe illness.

Studies of arbitrary combinations of antioxidants teach us little, since they cannot be compared or pooled and drawing conclusions about a specific antioxidant is impossible. Instead, factorial trials such as the one shown in Table 3 are informative regarding individual effects and possible interactions. Although the dose of vitamin C may influence the size of effect, it is likely that the baseline level of vitamin C also has an impact on the benefits of vitamin C administration and should therefore be measured. In addition, it is important that any further studies are sufficiently powered to be able to detect a reasonable effect and the required sample size should be calculated accordingly.

Vitamin C costs only pennies per gram, whereas one day in the ICU may cost thousands of dollars; therefore, an 8% decrease in ICU stay from the administration of 2 g/day of vitamin C warrants further research.

## Figures and Tables

**Table 1:** Characteristics of included trials.

Trial [Reference]	Country	Settings	*N*	Route	Vitamin C Administration		Control Group Time	
					Dose (g/day)	Duration (days)	ICU Stay (days)	Ventilation (hours)
Mirmohammadsadeghi 2018 [121]	Iran	Cardiac	314	iv	1	4	2.2	
Donovan 2012 [112]	USA	Cardiac	304	po	2	5	1.9	
Bjordahl 2012 [111]	USA	Cardiac	185	po	2	5	4.3	33.6
Papoulidis 2011 [110]	Greece	Cardiac	170	iv	1	5	2.1	
Sarzaeem 2014 [116]	Iran	Cardiac	170	iv	1	5	3.0	
Amini 2018 [120]	Iran	Cardiac	138	po	3	2	2.3	6.7
Antonic 2017 [119]	Slovenia	Cardiac	105	iv	2	5	1.3	
Alsfahey 2017 [118]	Egypt	Cardiac	100	po	2	1	3.6	
Dehghani 2014 [113]	Iran	Cardiac	100	po	1	5	2.1	15.4
Eslami 2007 [108]	Iran	Cardiac	100	po	2	5	2.6	
Dingchao 1994 [106]	China	Cardiac	85	iv	17 *	1	1.9	
Abdoulhossein 2018 no vitE [122]	Iran	Lung contusion	40	iv	0.5	2	5.2	
Abdoulhossein 2018 vitE [122]	Iran	Lung contusion	40	iv	0.5	2	5.2	
Ebade 2014 [114]	Egypt	Cardiac	40	iv	3	5	3.2	2.0
Tanaka 2000 [107]	Japan	Burns	37	iv	110 *	1		511
Zabet 2016 [117]	Iran	Sepsis	28	iv	7 *	3	20.6	46.8
Colby 2011 [109]	USA	Cardiac	24	po	1	5	2.0	
Fowler 2014 [115]	USA	Sepsis	24	iv	3.5–14 *	4	11	

The trials are listed by the number of patients (N). Mean age and proportion of males are shown in Supplementary file S2. *, calculated for the weight of 70 kg. Abbreviations: po, per oral; iv, intravenous.

**Table 2:** Meta-analyses of all included trials and selected subgroups on the length of ICU stay.

Selection of Trials	*N* Trials	*N* Patients	Estimate of Effect			Heterogeneity	
			RoM	95% CI	*p*	*I* ^2^	*p*
All	17	1967	0.835	0.81–0.86	10^−26^	90%	10^−24^
Exclusion1 ^(a)^	16	1882	0.908	0.87–0.94	10^−6^	58%	0.002
Exclusion2 ^(a)^	15	1842	0.923	0.89–0.96	0.00003	35%	0.088
**Final meta-analysis ^(b)^**	**12**	**1766**	**0.922**	**0.89–0.96**	**0.00003**	**43%**	**0.055**
Sensitivity analysis ^(c)^	9	1312	0.927	0.88–0.98	0.005	51%	0.036
Cardiac trials	11	1726	0.918	0.88–0.95	0.00001	43%	0.066
Oral vitamin C	6	927	0.914	0.86–0.97	0.003	49%	0.08
Intravenous vitamin C	6	839	0.928	0.88–0.97	0.003	47%	0.10
Trials in Iran	6	862	0.927	0.88–0.98	0.005	43%	0.12
Trials out of Iran	6	904	0.917	0.87–0.97	0.002	53%	0.06
1-2 days ICU ^(d)^	7	1231	0.943	0.89–0.99	0.027	52%	0.05
3-5 days ICU ^(d)^	5	535	0.899	0.85–0.95	0.0001	23%	0.3

^(a)^ Exclusion1 is the meta-analysis in which the Dingchao trial [106] is excluded. Exclusion2 is the meta-analysis in which the Abdoulhossein study vitamin E patients [122] is further excluded. ^(b)^ The final meta-analysis excludes the three small trials by Colby [109], Fowler [115], and Zabet [117] in addition to the two trials excluded in Exclusion2. Note that the exclusion of these three small trials has no meaningful effect on the point estimate of effect (RoM: 0.9227 versus 0.9220) or on the *p*-value, but their exclusion increases the level of heterogeneity from *I*^2^ = 35% to 43%. The Final meta-analysis, indicated by bold, is shown as Figure 3. The subgroup analyses below the final meta-analysis are based on the final meta-analysis. ^(c)^ In the sensitivity analysis, we excluded two trials with no description of allocation [114,118], and a quasi-randomized trial [121]. ^(d)^ Length of ICU stay in the control group of the trial; see the comparison of the subgroups in Supplementary file S1. Abbreviations: RoM, ratio of means; for example, RoM = 0.922 of the final model indicates that vitamin C shortens the mean length of ICU stay by 7.8%.

**Table 3:** Interaction between vitamin C and vitamin E in the Abdoulhossein (2018) trial.

Vitamin E	Vitamin C		Difference (95% CI)
	No	Yes	
No	5.2 (1.67)	5.5 (1.73)	+0.3 (−0.8 to +1.4)
Yes	5.2 (1.74)	3.5 (0.5)	−1.7 (−2.5 to −0.9)

Abdoulhossein administered 0.5 g/day of vitamin C and/or 1 g/day of vitamin E intravenously to lung contusion patients [122]. The length of ICU stay is in days, with SD in the parentheses; N = 20 in each group. When the interaction term was added after the vitamin C and vitamin E terms, the model was improved by *χ*^2^ (1 df) = 8.83 corresponding to *p* = 0.004 in the analysis of variance. Within the vitamin E patients, the difference between the vitamin C and no-vitamin C groups was highly significant (*p* < 0.0001). See Supplementary files S1 and S2 for the calculations. Although the statistical evidence for the interaction between vitamins C and E is very strong in this table, the methods were not reported in sufficient detail. We were unable to contact Dr. Abdoulhossein via emails to ask for the details. Thus, these findings should be considered cautiously, even though it is evident that this pattern of findings encourages further studies to use 2 × 2 factorial designs. When interaction is possible, there is much more information available from a trial using a factorial design than from a trial in which there are two or three parallel groups.

## Supplementary Materials

The following are available online at https://www.mdpi.com/2072-6643/11/4/708/s1, Supplementary file S1 (PDF): Table S1: Descriptions of included trials, Figure S1: Forest plot for all 17 trials for the length of ICU stay; printouts of statistical analyses. Supplementary file S2 (XLSX): spreadsheet showing the extracted data and calculations.
