# Supplementary material for: Vitamin C Can Shorten the Length of Stay in the ICU: A Meta-Analysis
Source: Nutrients. 2019 Mar 27;11(4):708. doi: 10.3390/nu11040708 (PMC6521194; doi:10.3390/nu11040708)
Supplement: Supplementary file 1 [file nutrients-11-00708-s001.zip › Supplement_S1_Hemila_2019_3_24.pdf]

Vitamin C can shorten the length of stay in the ICU: a meta-analysis

Harri Hemilä and Elizabeth Chalker

Supplementary file S1

to a paper published in **Nutrients**
https://www.mdpi.com/journal/nutrients

version 2019-3-24

Harri Hemilä
Department of Public Health, POB 20
University of Helsinki, Finland
E-mail: harri.hemila@helsinki.fi
Home page: http://www.mv.helsinki.fi/home/hemila

Contents

Fig. S1: Forest plot for all 17 trials for the length of ICU stay	2
Final meta-analysis on ICU stay	3
Meta-analysis by the length of ICU stay in the control group	4
Calculation of interaction P-value in the Abdoulhossein trial	5
Modification of vitamin C effect on mechanical ventilation by the duration of mechanical ventilation in control group	7
Effect on mechanical ventilation in non-cardiac trials	8
Other effects: Zabet (2016) trial mortality	9
Table S1: Descriptions of included trials	10

Fig. S1: Forest plot for all 17 trials for the length of ICU stay

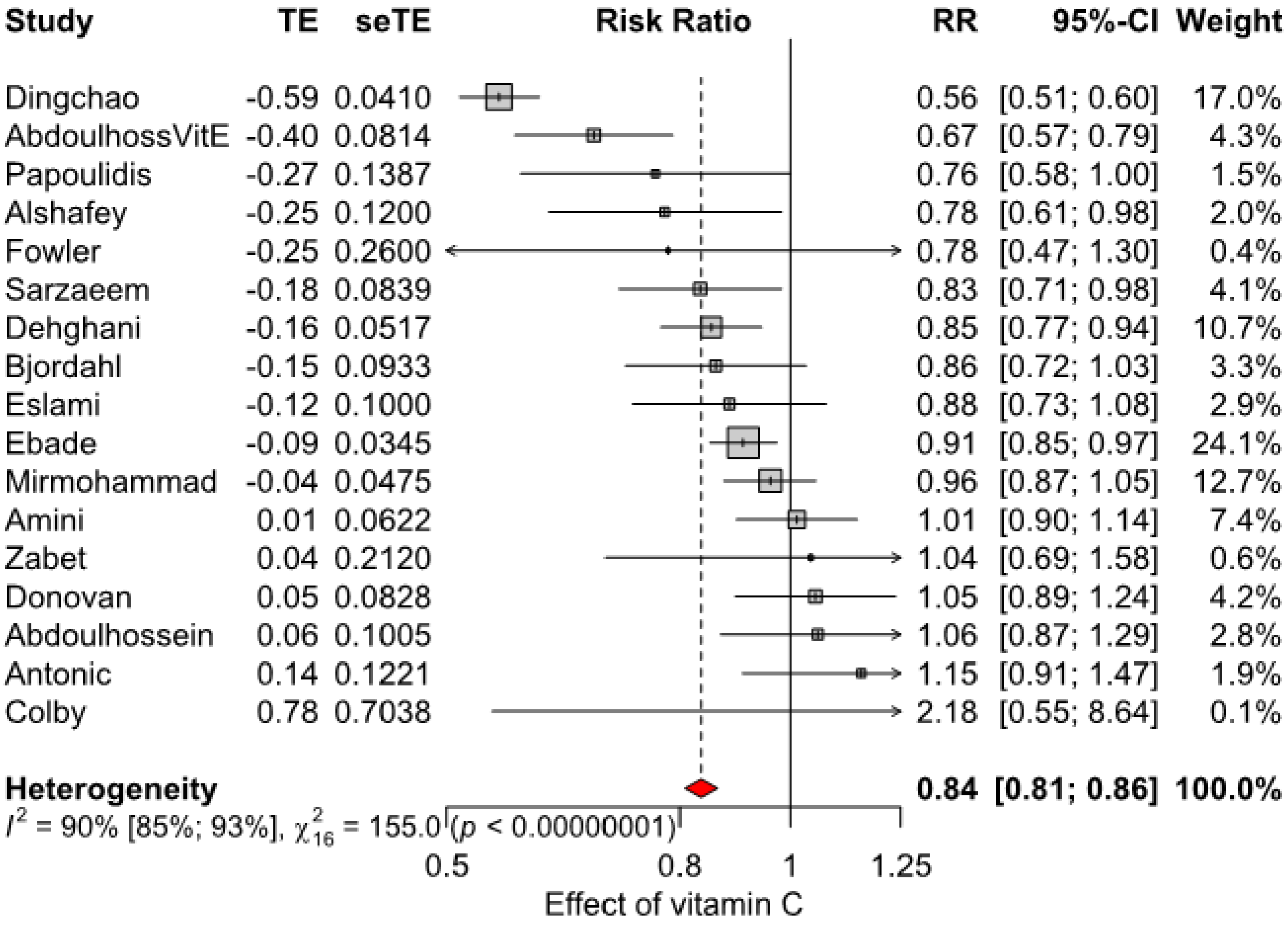


```
> summary(icuMA)
Number of studies combined: k = 17

                       RR             95%-CI       z  p-value
Fixed effect model 0.8345 [0.8072; 0.8627] -10.67 < 0.0001

Quantifying heterogeneity:
tau^2 = 0.0458; H = 3.11 [2.58; 3.74]; I^2 = 89.7% [85.0%; 92.9%]

Test of heterogeneity:
      Q d.f.  p-value
 154.60   16 < 0.0001

Details on meta-analytical method:
- Inverse variance method
```

Final meta-analysis on ICU stay

```
Number of studies combined: k = 12

                         RR            95%-CI     z  p-value
Fixed effect model 0.9220 [0.8879; 0.9576] -4.21 < 0.0001

Quantifying heterogeneity:
tau^2 = 0.0037; H = 1.33 [1.00; 1.86]; I^2 = 43.1% [0.0%; 71.1%]

Test of heterogeneity:
     Q d.f. p-value
 19.35   11  0.0551

Results for subgroups (fixed effect model):
              k     RR            95%-CI    Q  tau^2   I^2
Intravenous   6 0.9276 [0.8836; 0.9737] 9.35 0.0041 46.5%
Oral          6 0.9137 [0.8604; 0.9703] 9.85 0.0059 49.2%

Test for subgroup differences (fixed effect model):
                   Q d.f. p-value
Between groups  0.15    1  0.7024
Within groups  19.20   10  0.0378

Details on meta-analytical method:
- Inverse variance method
```

Meta-analysis by the length of ICU stay in the control group

days3=0 indicates that control group length of ICU stay was 1-2 days (7 trials)
days3=1 indicates that control group length of ICU stay was 3-5 days (5 trials)

```
Number of studies combined: k = 12

                       RR            95%-CI     z  p-value
Fixed effect model 0.9220 [0.8879; 0.9576] -4.21 < 0.0001

Quantifying heterogeneity:
tau^2 = 0.0037; H = 1.33 [1.00; 1.86]; I^2 = 43.1% [0.0%; 71.1%]

Test of heterogeneity:
     Q d.f.  p-value
 19.35   11   0.0551

Results for subgroups (fixed effect model):
          k     RR            95%-CI     Q  tau^2   I^2
days3=0   7 0.9431 [0.8954; 0.9934] 12.55 0.0058 52.2%
days3=1   5 0.8989 [0.8507; 0.9498]  5.26 0.0019 23.9%

Test for subgroup differences (fixed effect model):
                    Q d.f.  p-value
Between groups   1.55    1   0.2136
Within groups   17.80   10   0.0584

Details on meta-analytical method:
- Inverse variance method
```

The 5 trials in which control group ICU duration was 3-5 days

```
                  RR            95%-CI %W(fixed)
Alshafey      0.7765 [0.6138; 0.9825]       5.5
Sarzaeem      0.8334 [0.7070; 0.9823]      11.2
Bjordahl      0.8604 [0.7167; 1.0331]       9.1
Ebade         0.9095 [0.8500; 0.9731]      66.4
Abdoulhossein 1.0577 [0.8686; 1.2880]       7.8

Number of studies combined: k = 5

                       RR            95%-CI     z p-value
Fixed effect model 0.8989 [0.8507; 0.9498] -3.79  0.0001

Quantifying heterogeneity:
tau^2 = 0.0019; H = 1.15 [1.00; 1.80]; I^2 = 23.9% [0.0%; 69.0%]

Test of heterogeneity:
    Q d.f.  p-value
 5.26    4  0.2621

Details on meta-analytical method:
- Inverse variance method
```

Calculation of interaction P-value in the Abdoulhossein trial

https://doi.org/10.1016/j.amsu.2018.10.026
https://www.ncbi.nlm.nih.gov/pmc/articles/PMC6240669/

Test of interaction between vitamin C and vitamin E was calculated by two approaches.

1) Four groups of 20 observation were generated so that the means and SDs were identical with the four groups reported by Abdouhossein. This is described in sheet "Abdoul 2x2 analysis" of Supplementary file S2.
The interaction between vitamins C and E was calculated with linear modelling by R program package. The printout of this approach is shown on the next page

2) The interaction test was calculated with numerical calculation from the reported means and SDs. This is described in sheet "Abdoul ANOVA" of Supplementary file S2.

This is description of the four study groups generated for the calculation
See the generation in Supplementary file S2 (sheet "Abdoul 2x2 analysis")

```
> numSummary(Ax2[Ax2$vitC==0&Ax2$vitE==0,"days"], statistics=c("mean", "sd"))
 mean       sd  n
  5.2 1.670001 20
> numSummary(Ax2[Ax2$vitC==1&Ax2$vitE==0,"days"], statistics=c("mean", "sd"))
 mean       sd  n
  5.5 1.730001 20
> numSummary(Ax2[Ax2$vitC==0&Ax2$vitE==1,"days"], statistics=c("mean", "sd"))
 mean       sd  n
  5.2 1.740001 20
> numSummary(Ax2[Ax2$vitC==1&Ax2$vitE==1,"days"], statistics=c("mean", "sd"))
 mean        sd  n
  3.5 0.5000044 20
```

This is model for the vitamin C and vitamin E effects **without** interaction

```
Alm <- lm(Ax2$days ~ Ax2$vitC + Ax2$vitE)

Coefficients:
            Estimate Std. Error t value Pr(>|t|)
(Intercept)   5.7000     0.3059  18.634  < 2e-16 ***
Ax2$vitC     -0.7000     0.3532  -1.982  0.05107 .
Ax2$vitE     -1.0000     0.3532  -2.831  0.00591 **

Residual standard error: 1.58 on 77 degrees of freedom
Multiple R-squared:  0.1343,   Adjusted R-squared:  0.1118
F-statistic: 5.972 on 2 and 77 DF,  p-value: 0.003882
```

This is model for the vitamin C and vitamin E effects **with** interaction

```
AlmI <- lm(Ax2$days ~ Ax2$vitC*Ax2$vitE)

Coefficients:
                    Estimate Std. Error t value Pr(>|t|)
(Intercept)        5.200e+00  3.365e-01  15.452  < 2e-16
Ax2$vitC           3.000e-01  4.759e-01   0.630  0.53034
Ax2$vitE          -1.500e-07  4.759e-01   0.000  1.00000
Ax2$vitC:Ax2$vitE -2.000e+00  6.730e-01  -2.972  0.00396

Residual standard error: 1.505 on 76 degrees of freedom
Multiple R-squared:  0.2244,   Adjusted R-squared:  0.1938
F-statistic: 7.329 on 3 and 76 DF,  p-value: 0.0002221
```

These sum of squares and the F-value below are identical with those calculated from the published SD values in Supplementary file S2 (sheet "Abdoul ANOVA")

```
> anova(Alm,AlmI)
Analysis of Variance Table

Model 1: Ax2$days ~ Ax2$vitC + Ax2$vitE
Model 2: Ax2$days ~ Ax2$vitC * Ax2$vitE
  Res.Df    RSS Df Sum of Sq      F   Pr(>F)
1     77 192.13
2     76 172.13  1        20 8.8306 0.003965 **
```

Modification of vitamin C effect on mechanical ventilation by the duration of mechanical ventilation in control group

```
The analysis on the top compares the long vs short ventilation as two groups:
> venMA <- metagen(TE, seTE,
studlab,byvar=LongVenti,print.byvar=F,data=Ven,sm="RR", comb.random=F)
> venMA
             RR            95%-CI %W(fixed)            LongVenti
Tanaka   0.5681 [0.3548; 0.9095]       2.3 >24 hour ventilation
Zabet    0.7830 [0.6057; 1.0122]       7.7 >24 hour ventilation
Bjordahl 0.8571 [0.7444; 0.9868]      25.7 >24 hour ventilation
Dehghani 0.8682 [0.6554; 1.1502]       6.4 <24 hour ventilation
Ebade    1.0251 [0.9261; 1.1347]      49.5 <24 hour ventilation
Amini    1.0974 [0.8577; 1.4039]       8.4 <24 hour ventilation

Number of studies combined: k = 6

                        RR            95%-CI     z p-value
Fixed effect model 0.9412 [0.8764; 1.0109] -1.66  0.0965

Quantifying heterogeneity:
tau^2 = 0.0150; H = 1.59 [1.01; 2.49]; I^2 = 60.4% [2.8%; 83.8%]

Test of heterogeneity:
     Q d.f. p-value
 12.62    5  0.0273

Results for subgroups (fixed effect model):
                       k     RR            95%-CI    Q  tau^2    I^2
>24 hour ventilation   3 0.8185 [0.7263; 0.9224] 2.84 0.0072 29.6%
<24 hour ventilation   3 1.0172 [0.9305; 1.1119] 1.60      0   0.0%

Test for subgroup differences (fixed effect model):
                  Q d.f.  p-value
Between groups 8.17    1  0.0043
Within groups  4.44    4  0.3493

Details on meta-analytical method:
- Inverse variance method
>
The analysis at the bottom is metaregression, Logventi = Log(ventilation time):

> MetaRegression <- metareg(venMA,LogVenti)
> MetaRegression

Mixed-Effects Model (k = 6; tau^2 estimator: DL)

tau^2 (estimated amount of residual heterogeneity):     0 (SE = 0.0097)
tau (square root of estimated tau^2 value):             0
I^2 (residual heterogeneity / unaccounted variability): 0.00%
H^2 (unaccounted variability / sampling variability):   1.00
R^2 (amount of heterogeneity accounted for):            100.00%

Test for Residual Heterogeneity:
QE(df = 4) = 2.2034, p-val = 0.6984

Test of Moderators (coefficient(s) 2):
QM(df = 1) = 10.4118, p-val = 0.0013

Model Results:

          estimate      se     zval    pval    ci.lb    ci.ub
intrcpt     0.1013  0.0620   1.6339  0.1023  -0.0202   0.2228
LogVenti   -0.1845  0.0572  -3.2267  0.0013  -0.2966  -0.0725  **
```

Effect of vitamin C on mechanical ventilation in non-cardiac trials

```
> venMA3 <- metagen(TE, seTE, studlab,data=Ven3,sm="RR", comb.random=F)
> venMA3
           RR            95%-CI %W(fixed)
Tanaka 0.5681 [0.3548; 0.9095]      22.9
Zabet  0.7830 [0.6057; 1.0122]      77.1

Number of studies combined: k = 2

                        RR            95%-CI     z p-value
Fixed effect model 0.7274 [0.5807; 0.9114] -2.77  0.0057

Quantifying heterogeneity:
tau^2 = 0.0141; H = 1.17; I^2 = 27.4%

Test of heterogeneity:
    Q d.f. p-value
 1.38    1  0.2407

Details on meta-analytical method:
- Inverse variance method
> ((1-0.7274)*100)      # CHANGE TO THE %-SCALE
[1] 27.26
> ((1-0.5807)*100)
[1] 41.93
> ((1-0.9114)*100)
[1] 8.86
```

Other effects: Zabet (2016) trial mortality

```
> Zabet <- matrix(c(5,12,9,2),nrow=2)
> Zabet
     [,1] [,2]
[1,]    5    9
[2,]   12    2
> riskratio(Zabet)
$`data`
          Outcome
Predictor  Disease1 Disease2 Total
  Exposed1        5        9    14
  Exposed2       12        2    14
  Total          17       11    28

$measure
          risk ratio with 95% C.I.
Predictor   estimate      lower    upper
  Exposed1 1.0000000         NA       NA
  Exposed2 0.2222222 0.05811899 0.849683

$p.value
          two-sided
Predictor   midp.exact fisher.exact chi.square
  Exposed1          NA           NA         NA
  Exposed2 0.009822866   0.01830664 0.00675533
```

Table S1: Descriptions of included trials

Abdoulhossein 2018 no vitE

**Methods**	Double-blind, randomized placebo-controlled trial, February 2015 to June 2018. This is participants who were not administered vitamin E.<br>https://doi.org/10.1016/j.amsu.2018.10.026<br>https://www.ncbi.nlm.nih.gov/pmc/articles/PMC6240669/<br>https://www.ncbi.nlm.nih.gov/pubmed/30479762
**Participants**	Iran, severe blunt chest trauma, 26 M / 14 F; mean age: 40 yr (SD 9 yr); 20 vit C / 20 placebo.<br><br>**Inclusion:** Unilateral chest injury, being in serious to severer traumatic condition, although we tried to select the injured patients to be more isolated, however in 32% patients, had minor associated injuries<br><br>**Exclusion:** Penetrative trauma, patients in critical condition, complicated patients, needing intubation more than 3 d, emergency surgical intervention, opium addiction, COPD, asthma, Glasgow Coma Scale (GCS) less than 13, major associated trauma, pulmonary edema, blood transfusion and thromboembolic effects in long bone fracture, pneumonia, ARDS, combined chest wall Injuries with lung contusion, unilateral flail segment <4 ribs, hemothorax drained with chest tube.
**Interventions**	**Vitamin C group:** IV vitamin C (500 mg in 50 ml of normal saline).<br>The report states "The trial process was done during 24-48 h of ICU admission" (p 154), but there is no description whether the infusion was only one time or on two days.<br>**Control group:** IV distilled water (5 ml in 50 ml normal saline)
**Outcomes**	Length of ICU stay measured after extubation (p 153)
**Notes**	Poorly written report, but no obvious methodological shortcomings.<br><br>We tried to contact the first author to ask for methodological details (email 2018-12-11, 2018-12-29 and 2019-1-8), and we sent an email to all five authors (2019-1-19). We did not receive any responses

Risk of bias table

Bias	Authors' judgement	Support for judgement
Random sequence generation (selection bias)	Low risk	"Randomization assignment by permuted blocked method was conducted with using computer-generated random numbers." (p 153)
Allocation concealment (selection bias)	Low risk	"Randomization and allocation concealment were conducted by the researchers and participants and were carried out by a trained staff at the clinic. Another person, who was not involved in the trial and not aware of random sequences, assigned the subjects for taking supplements" (p 153)
Blinding of participants and personnel (performance bias)	Low risk	"All critical care treatment decisions including tracheal intubation, mechanical ventilation if need, extubation, length of ICU staying with prospect of intention to treatment, under the direction of thoracic surgeon and anesthesiologist, who was not aware from protocol, was done." (p 153)
Blinding of outcome assessment (detection bias)	Low risk	"double-blind" (Abstract), but no clear description in Methods section
Incomplete outcome data (attrition bias)	Unclear risk	No description whether the 40 reported patients equals the number of randomized participants, no flow diagram

Abdoulhossein 2018 yes vitE

**Methods**	Double-bline, randomized placebo-controlled trial, February 2015 to June 2018. This is participants who were administered vitamin E.<br>https://doi.org/10.1016/j.amsu.2018.10.026<br>https://www.ncbi.nlm.nih.gov/pmc/articles/PMC6240669/<br>https://www.ncbi.nlm.nih.gov/pubmed/30479762
**Participants**	Iran, severe blunt chest trauma, 28 M / 12 F; mean age: 40 yr (SD 9 yr); 20 vit C / 20 placebo.<br><br>**Inclusion:** Unilateral chest injury, being in serious to severer traumatic condition, although we tried to select the injured patients to be more isolated, however in 32% patients, had minor associated injuries<br><br>**Exclusion:** Penetrative trauma, patients in critical condition, complicated patients, needing intubation more than 3 d, emergency surgical intervention, opium addiction, COPD, asthma, Glasgow Coma Scale (GCS) less than 13, major associated trauma, pulmonary edema, blood transfusion and thromboembolic effects in long bone fracture, pneumonia, ARDS, combined chest wall Injuries with lung contusion, unilateral flail segment <4 ribs, hemothorax drained with chest tube.
**Interventions**	Both of these study groups were administered vitamin E: "intravenous infusion vitamin E (1000 IU in 50 ml of emulsion)"<br>**Vitamin C group:** IV vitamin C (500 mg in 50 ml of normal saline).<br>The report states "The trial process was done during 24-48 h of ICU admission" (p 154), but there is no description whether the infusion was only one time or on two days.<br>**Control group:** IV distilled water (5 ml in 50 ml normal saline)
**Outcomes**	Length of ICU stay measured after extubation (p 153)
**Notes**	Poorly written report, but no obvious methodological shortcomings. We excluded the study from our final ICU meta-analysis.<br>We tried to contact the first author to ask for methodological details (email 2018-12-11, 2018-12-29 and 2019-1-8), and we sent an email to all five authors (2019-1-19). We did not receive any responses

Risk of bias table

Bias	Authors' judgement	Support for judgement

Random sequence generation (selection bias)	Low risk	"Randomization assignment by permuted blocked method was conducted with using computer-generated random numbers." (p 153)
Allocation concealment (selection bias)	Low risk	"Randomization and allocation concealment were conducted by the researchers and participants and were carried out by a trained staff at the clinic. Another person, who was not involved in the trial and not aware of random sequences, assigned the subjects for taking supplements" (p 153)
Blinding of participants and personnel (performance bias)	Low risk	"All critical care treatment decisions including tracheal intubation, mechanical ventilation if need, extubation, length of ICU staying with prospect of intention to treatment, under the direction of thoracic surgeon and anesthesiologist, who was not aware from protocol, was done." (p 153)
Blinding of outcome assessment (detection bias)	Low risk	"double-blind" (Abstract), but no clear description in Methods section
Incomplete outcome data (attrition bias)	Unclear risk	No description whether the 40 reported patients equals the number of randomized participants, no flow diagram

Alshafey 2017

**Methods**	Randomized trial, between 2011 and 2016<br>https://doi.org/10.1016/j.jescts.2017.04.003
**Participants**	Egypt. CABG patients, 57 M / 43 F. mean age: 55 yr (SD 6 yr), 50 vit C / 50 control.<br><br>**Inclusion:** "elective cases of CABG, with ejection fraction (EF) above 30%, with one to four grafts"<br><br>**Exclusion:** "without other cardiac disease (rheumatic, congenital, etc ….) and without ischemic mitral regurgitation. We excluded the patients who underwent CABG before, emergency cases of CABG, patients with EF less than 30%, and patients having contraindications to b - blocker or ascorbic acid"
**Interventions**	**Vit C before the operation:**<br>**Dose:** 2 g/d "ascorbic acid (2 g-daily)."<br>**Method:** not described explicitly, but apparently oral administration<br>**Timing:** "for at least three days pre-operatively"<br>**Vit C after operation:**<br>**Dose:** no description, apparently vitamin C was not continued after operation.<br>We count that the duration of vitamin C administration continues for the day of surgery and treandfer to the ICU, ie. 1 day<br>**Control group:** no placebo tablets.
**Outcomes**	Length of ICU stay
**Notes**	Very poorly written report. In a sensitivity analysis we excluded the study.<br><br>The first page of the study report has the email addresses of all four authors. We tried to contact them to ask for methodological details (email 2018-12-29, 2019-1-8 and 2019-1-19). We did not receive any responses.<br><br>Alshafey reported ICU duration in vitamin C group 2.78 (SD 1.33) d and in control group 3.58 (SD 0.73) d.<br><br>Calculation of the P-value with the Taylor series formula from these reported mean and SD-values gives P = 0.0006.<br><br>However, Alshafey reported P = 0.035 for the effect of vitamin C on ICU stay. To be conservative, we used this P = 0.035 to calculate the consistent SE(log(RoM)), see Supplementary file 2.<br><br>We cannot conclude whether there are errors eg in the

reported SD-values. In sensitivity analysis we excluded the study.

Alshafey reported the duration of ventilation as <24 hr and >24 hr, and the rate of long ventilation (>24 hr) in the vitamin C group was 28% (14/50) and in the control group 34% (17/50). This gives RR = 0.824 which is consistent with vitamin C being beneficial. However, for the comparison of the 14/36 vs. 17/33 table, Alshafey reported P = 0.048 (their Table 7) which obviously is erroneous. The Fisher exact test gives P = 0.66 for the 14/36 vs. 17/33 table.

We tried to contact Alsfahey and colleagues, but we were unsuccessful (see above). We had hoped to ask whether they have the continuous data for the duration of ventilation, so that we could include the study in our analysis. We excluded the study from the analysis of duration of ventilation.

Risk of bias table

Bias	Authors' judgement	Support for judgement
Random sequence generation (selection bias)	Unclear risk	Abstract states "This prospective randomized study" (p 198). However, the Methods section does not give any information about allocation. Given that 100 participants were allocated to vitamin C and control groups, the resulting exact 50-50 split suggests the authors might have used alternative allocation. Even if that was the case, we do not consider there is high risk of bias. The two groups are well balanced for variables described in their Table 1 (p 200).
Allocation concealment (selection bias)	Unclear risk	No descriptions
Blinding of participants and personnel (performance bias)	Unclear risk	No descriptions
Blinding of outcome assessment (detection bias)	Unclear risk	No descriptions
Incomplete outcome data (attrition bias)	Unclear risk	No description whether the 100 reported patients equals the number of randomized participants, no flow diagram

Amini 2018

**Methods**	Randomized trial with 4-arms; we restrict to vitamin C and control arms<br>https://doi.org/10.21470/1678-9741-2017-0071<br>https://www.ncbi.nlm.nih.gov/pmc/articles/PMC5985838<br>https://www.ncbi.nlm.nih.gov/pubmed/29898141
**Participants**	Iran, CABG patients, 57 M / 51 F; mean age: 60 yr (SD 10 yr); 67 vit C / 71 control.<br><br>**Inclusion:** NYHA class of I-III undergoing elective off-pump CABG. Patients underwent standard intravenous anesthesia and were transferred to ICU for post-cardiac surgery for further monitoring and recovery.<br><br>**Exclusion:** Change from off-pump to on-pump surgery, known drug allergy, history of COPD, anemia, congestive heart failure (CHF), active sepsis, preoperative ejection fraction lower than 40%, preoperative creatinine above 1.3 mg/dL, use of nephrotoxic drugs, coronary angiography, intraoperative transfusion of more than 2 units of red blood cells, perioperative use of intra-aortic balloon counterpulsation, perioperative requirement for high-dose vasopressors, and any intraoperative life-threatening events such as fatal arrhythmias, excessive bleeding, or desaturation.r
**Interventions**	**Vit C before the operation:**<br>**Dose:** 3 g/d "vitamin C... 1500 mg ... tablets, twice a day, from 24 hours before the operation until two postoperative days."<br>**Method:** po<br>**Timing:** "24 h before operation"<br>**Vit C after operation:**<br>**Dose:** 3 g/d "1500 mg .. tablets ... twice a day"<br>**Method:** po<br>**Duration:** 2 d "until two postoperative days"<br>**Control group:** no tablets.
**Outcomes**	Length of ICU stay<br>Length of mechanical ventilation
**Notes**	"Ventilation times were 7.33±6.02, 10.68±27.15, 5.90±3.13, and 25.36±157.5 hours in the vitamin C, NAC, selenium and control groups, respectively (P=0.429). The mean overall ICU LOS was 2.66±3.87 days with a median of 2 days. The ICU LOS in the vitamin C, NAC, selenium and control groups was 2.36±0.86, 2.57±1.50, 2.30±0.78, and 3.20±7.36 days, respectively (P=0.207)." (p 131)<br><br>Thus, the SD for both ventilation and ICU LOS was much

greater than the SD for each of the other 3 groups.

We were able to contact Dr. Amini and he kindly sent us their data set (email 2018-11-6). We found that there was one patient (#198) in the control group who had ICU LOS 64 days and ventilation time 1333 hours. We removed this outlier and calculated that mean ICU LOS was 2.329 (SD 0.847) days and mean ventilation time 6.68 (SD 4.26) hours in the control group. These revised SD values are closely similar to the SD values of the three other arms, compare above.

We were also able to get more information of the Methods from Dr. Amini (email 2018-12-13), see below.

Risk of bias table

Bias	Authors' judgement	Support for judgement
Random sequence generation (selection bias)	Low risk	"Using a computer-based randomization method" (p 130)
Allocation concealment (selection bias)	Low risk	"The patients, the researcher who collected the data and the one who analyzed the data were blinded to the study. However, the physicians and nurses in charge were aware of the allocated groups" (email 2018-12-13)
Blinding of participants and personnel (performance bias)	Low risk	"The patients, the researcher who collected the data and the one who analyzed the data were blinded to the study. However, the physicians and nurses in charge were aware of the allocated groups" (email 2018-12-13)<br><br>Given that no benefit of vitamin C was found from vitamin C, we do not consider that this lack of benefit could be biased by "the physicians and nurses in charge were aware of the allocated groups". Therefore, we classify this as low risk for personnel.
Blinding of outcome assessment (detection bias)	Low risk	"The patients, the researcher who collected the data and the one who analyzed the data were blinded to the study. However, the physicians and nurses in charge were aware of the allocated groups" (email 2018-12-13)<br><br>Given that no benefit of vitamin C was found from vitamin C, we do not consider that this lack of benefit could be biased by "the physicians and nurses in charge were aware of the allocated groups". Therefore, we classify this as low risk for outcome assessment.
Incomplete outcome data (attrition bias)	Low risk	6 vitamin C participants and 2 control participants discontinued (p 131; Fig 1) and we (HH and EC) removed 1 control participant, see Notes above.

Antonic 2016

**Methods**	Randomized trial, March 2013 to June 2014.<br>http://dx.doi.org/10.1016/j.jjcc.2016.01.010<br>https://www.ncbi.nlm.nih.gov/pubmed/26917198
**Participants**	Slovenia, CABG patients, 82 M / 23 F; mean 64 yr (SD 12 yr); 52 vit C / 53 control.<br><br>**Inclusion:** Patients who were scheduled for an elective CABG.<br><br>**Exclusion:** Emergency operations, any concomitant valve or other surgery, preoperative history of AF, permanent pacemaker, hyperoxaluria or history of nephrolithiasis, and off-pump surgery.
**Interventions**	**Vit C before the operation:**<br>**Dose:** 4 g/day<br>**Method:** iv<br>**Timing:** "2 g of ascorbic acid 24 h and 2 h prior to surgery"<br>**Vit C after operation:**<br>**Dose:** 2 g/d "1 g twice a day for five days after the surgery"<br>**Method:** iv<br>**Duration:** 5 d<br>**Placebo** was not used, but other iv medications serve as the placebo
**Outcomes**	Length of ICU stay
**Notes**	Additional information was received by emails from Miha Antonic on 2016-12-2, see below.<br>The calculation of sample size in the trial was not correct, see:<br>http://dx.doi.org/10.1016/j.jjcc.2016.10.010<br>However, that problem does not challenge the internal validity of the trial.

Risk of bias table

Bias	Authors' judgement	Support for judgement
Random sequence generation (selection bias)	Low risk	"The enrolled patients were then randomly assigned to .." (text) and "For the randomization, we used the www.random.org online service" (Miha Antonic email 2016-12-1).
Allocation concealment (selection bias)	Low risk	"The randomization process was as follows: First, 52 As and 53 Bs were randomly arranged from 1 to 105" ... "the order of admittance of the patients to surgery was strictly in concordance with the Hospital's electronic waiting lists, which are under close surveillance of Ministry of Health and cannot be in any way manipulated" (Miha Antonic email 2016-12-1).
Blinding of participants and personnel (performance bias)	Low risk	"there was no blinding (and therefore use of placebo) in the study" (Miha Antonic email 2016-12-1).<br><br>Given that the findings of the study were negative, we do not consider that bias caused by lack or blinding is a reasonable explanation for the finding. In contrast, if the finding is positive, it is reasonable to speculate if the positive finding emerged from poor blinding. Therefore, we classify that there is low risk of bias in the negative result.
Blinding of outcome assessment (detection bias)	Low risk	Given that the findings of the study were negative, we do not consider that bias caused by lack or blinding is a reasonable explanation for the finding. In contrast, if the finding is positive, it is reasonable to speculate if the positive finding emerged from poor blinding. Therefore, we classify that there is low risk of bias in the negative result.
Incomplete outcome data (attrition bias)	Low risk	"there were no dropouts" (Miha Antonic email 2016-12-1).

Bjordahl 2012

**Methods**	Triple-blind, randomised placebo-controlled trial, October 2009 to April 2011.<br>http://dx.doi.org/10.1016/j.amjsurg.2012.03.012<br>http://www.ncbi.nlm.nih.gov/pubmed/23022248
**Participants**	USA, CABG patients, 124 M / 61 F; mean 63 yr (SD 12 yr); 89 vit C / 96 placebo.<br><br>**Inclusion:** >18 yr who were scheduled to undergo CABG.<br><br>**Exclusion:** current AF, temporary or permanent pacemaker, life expectancy <1 month, emergency surgery precluding the initiation of study protocol the evening before surgery, current pregnancy.
**Interventions**	**Vit C before the operation:**<br>**Dose:** 2 g<br>**Method:** po<br>**Timing:** "the evening before surgery"<br>**Vit C after operation:**<br>**Dose:** 2 g/d "1 gram twice daily"<br>**Method:** po<br>**Duration:** 5 d<br>**Placebo:** "identical placebo capsules at the same intervals"; "the inert substance for both treatment and placebo capsules was talc."
**Outcomes**	Length of ICU stay<br>Length of mechanical ventilation
**Notes**	"Both ascorbic acid and inert placebo capsules were prepared by a custom pharmacy" (p 863) indicates that the products were not commercial.<br>Bjorndahl reported the duration of ventilation in vitamin C group 1.2 (SD 0.8) d and in control group 1.4 (SD 1.0) d. Calculation of the P-value with the Taylor series formula from these reported mean and SD-values gives P = 0.13.<br>Bjorndahl reported P = 0.032 (Mann Whitney) for the effect of vitamin C on ICU stay. We used this P = 0.032 to calculate the consistent SE(log(RoM)), see Supplementary file.

Risk of bias table

Bias	Authors' judgement	Support for judgement
Random sequence generation (selection bias)	Low risk	"Enrolled participants were randomized to either ... The pharmacy department maintained the randomization list ..." (p 863).
Allocation concealment (selection bias)	Low risk	"The pharmacy department maintained the randomization list and assigned participants to the placebo and treatment arms of the study in a blinded fashion. Participants, clinicians, and evaluators were blinded to the treatment assignments and the blind was not broken until after data analyses were complete" (p 863).
Blinding of participants and personnel (performance bias)	Low risk	See above.
Blinding of outcome assessment (detection bias)	Low risk	See above.
Incomplete outcome data (attrition bias)	Low risk	13 participants were withdrawn from analysis because of surgery postponement/cancellation (3), presence of exclusion criteria at the time of enrollment (4), protocol violation (1), cardiac surgery without CABG (3), use of bloodless therapy for religious purposes (1) and intraoperative exsanguination and death unrelated to current study (1).

Colby 2011

**Methods**	Double-blind, randomised placebo-controlled trial, April 2009 to March 2010.<br>http://dx.doi.org/10.2146/ajhp100703<br>http://www.ncbi.nlm.nih.gov/pubmed/21856809
**Participants**	USA, CABG and valvular surgery patients, 19 M / 5 F, mean age: vitamin C 68 yr (SD 10 yr), placebo 62 yr (SD 7), 13 vit C / 11 placebo.<br><br>**Inclusion:** >18 yr who were scheduled to undergo CABG, valvular surgery, or both.<br><br>**Exclusion:** excluded if they were pregnant,had a history of renal calculi or had hypersenstivity to ascorbic acid.
**Interventions**	**Vit C before the operation:**<br>**Dose:** 2 g<br>**Method:** po<br>**Timing:** "night before surgery"<br>**Vit C after operation:**<br>**Dose:** 1 g/d "500 mg of oral ascorbic acid or matching placebo was given twice daily"<br>**Method:** po<br>**Duration:** 5 d<br>**Placebo:** "Both vit C and placebo were placed into identical capsules to allow for double-blinding."
**Outcomes**	Length of hospital stay
**Notes**	No reply to our emails.

Risk of bias table

Bias	Authors' judgement	Support for judgement
Random sequence generation (selection bias)	Low risk	"... randomized using a computer-generated sequence with a 1:1 allocation and a random block size of 10" (p 1633).
Allocation concealment (selection bias)	Low risk	"Study patients, cardiothoracic surgeons, caregivers, and investigators, including those responsible for data collection, were blinded to the treatment allocation" (p 1633).
Blinding of participants and personnel (performance bias)	Low risk	See above.<br><br>"Both the ascorbic acid and placebo were placed into identical capsules to allow for double-blinding." (p 1633)
Blinding of outcome assessment (detection bias)	Low risk	See above.
Incomplete outcome data (attrition bias)	Low risk	One patient "suffered a ventricular arrhythmia before undergoing cardiothoracic surgery and was excluded" (p 1634).

Dehghani 2014

**Methods**	Randomised trial, March 2012 to March 2013<br>http://www.ncbi.nlm.nih.gov/pubmed/24293167<br>http://dx.doi.org/10.5603/CJ.a2013.0154<br>http://czasopisma.viamedica.pl/cj/article/view/36075
**Participants**	Iran, CABG patients, 74 M / 26 F, mean age: 61 yr (SD 7 yr); 50 vit C / 50 control.<br><br>**Inclusion:** Patients who underwent elective isolated on-pump CABG surgery, age >50 yr, no history of CABG surgery, taking beta-blocker before and after surgery.<br><br>**Exclusion:** history of any cardiac arrhythmia and/or being under anti-arrhythmic therapy, being under digoxin therapy, having pacemaker, severe CHF and/or LVEF <30%, renal failure, severe hepatic failure, COPD, no occurrence of intra- or post-operative cardiopulmonary arrest, or any degree of cardiac blockade and/or bradycardia.
**Interventions**	**Vit C before the operation:**<br>**Dose:** 2 g "2 g of vitamin C tablets before the surgery"<br>**Method:** po<br>**Timing:** "All patients took the tablets within 12 hours before surgery" (email 2015-9-9)<br>**Vit C after operation:**<br>**Dose:** 1 g/d "500 mg twice daily"<br>**Method:** po<br>**Duration:** 5 d<br>**Placebo:** No placebo, but the patients received many drugs and it is unlikely that they identified vitamin C among all the other administered drugs. We classify that all the other drugs serve as a functional placebo to vitamin C.
**Outcomes**	Length of ICU stay<br>Length of mechanical ventilation
**Notes**	"Our study was funded by Urmia University of Medical Sciences, Iran." (email 2015-10-1)<br>Additional information was received by emails from Yousef Rezaei (2015-4-11, 2015-4-22 and 2015-9-9), see above and below.<br>Dehghani reported ICU duration in vitamin C group 1.79 (SD 0.313) d and in control group 2.10 (SD 0.61) d. Calculation of the P-value with the Taylor series formula from the reported mean and SD-values gives P = 0.0009. However, Dehghani reported P = 0.002 (Mann Whitney) for the effect of vitamin C on ICU stay. We used this P = 0.002 to calculate the consistent SE(log(RoM)), see Supplementary file 2.

Risk of bias table

Bias	Authors' judgement	Support for judgement
Random sequence generation (selection bias)	Low risk	"All patients were randomized into two groups in a 1:1 ratio using random-number table" (p 493).
Allocation concealment (selection bias)	Low risk	"Neither ward physician nor Holter interpreter were aware of the patients' group. Only one who analyzed data was aware of the patients' group" (email 2015-4-11) and<br><br>"we did not let ward physician and surgeons to know which of patients taking vitamin c or not, except for being informed about the conduction of our trial and prescribing some of patients to take vitamin c. Furthermore, patients were informed that they would be included in our trial to be prescribed vitamin c" (email 2015-4-22).
Blinding of participants and personnel (performance bias)	Low risk	"Neither ward physician ... were aware of the patients' group. " (email 2015-4-11) and<br><br>"we did not let ward physician and surgeons to know which of patients taking vitamin c or not, except for being informed about the conduction of our trial and prescribing some of patients to take vitamin c. Furthermore, patients were informed that they would be included in our trial to be prescribed vitamin c" (email 2015-4-22).
Blinding of outcome assessment (detection bias)	Low risk	See above.
Incomplete outcome data (attrition bias)	Low risk	"There was no patient withdrawal or missing during study. All allocated ones completed study" (email 2015-4-11 and 2015-10-1).

Dingchao 1994

**Methods**	Controlled trial<br>https://doi.org/10.1055/s-2007-1016504<br>https://www.ncbi.nlm.nih.gov/pubmed/7863489
**Participants**	China, cardiac surgery patients, ? M / ? F; mean age Not described; 45 vit C / 40 control.<br><br>**Inclusion:** Patients undergoing cardiopulmonary bypass (CPB)<br><br>**Exclusion:** Not described
**Interventions**	**Vit C before the operation:**<br>**Dose:** 0.125 g/kg "30 minutes before CPB"<br>**Method:** iv<br>**Timing:** 30 min before CPB<br>**Vit C at the aortic declamping:**<br>**Dose:** 0.125 g/kg "at the aortic declamping"<br>**Method:** iv<br>**Duration:** The 2 doses mentioned above with total 0.25 g/kg correspond to 17.5 g vitamin C for a 70 kg person in one day.<br>**Placebo** was
**Outcomes**	Length of ICU stay
**Notes**	Poorly reported study, but this is the oldest of the included. We excluded the study from our final ICU meta-analysis.

Risk of bias table

Bias	Authors' judgement	Support for judgement
Random sequence generation (selection bias)	Unclear risk	Not described. However, "there were no significant baseline differences in age, weight, CPB time, and aortic clamping time between the two groups" (p 276)
Allocation concealment (selection bias)	Unclear risk	Not described
Blinding of participants and personnel (performance bias)	Unclear risk	Not described
Blinding of outcome assessment (detection bias)	Unclear risk	Not described
Incomplete outcome data (attrition bias)	Unclear risk	Not described

Donovan 2012

**Methods**	Randomised placebo-controlled 2x2 factorial study with vitamin C and amiodarone, August 2009 to January 2012<br>https://clinicaltrials.gov/ct2/show/NCT00953212
**Participants**	USA, CABG patients, 230 M / 74 F, mean 65 yr (SD 9 yr); 150 vit C / 154 Placebo<br><br>**Inclusion:** >18 yr, all comers for elective or urgent open heart surgery (CABG, Valve repair or replacement, Combined CABG/Valves, CABG/other, Other)<br><br>**Exclusion:** history of AF, emergency surgery, contraindications to study medications, untreated thyroid disease, hepatic failure, pregnancy
**Interventions**	**Vit C before the operation:**<br>**Dose:** 2 g/d<br>**Method:** po<br>**Timing:** 2 g "evening before surgery" and 2 g "morning of surgery"<br>**Vit C after operation:**<br>**Dose:** 2 g/d<br>**Method:** po<br>**Duration:** 5 d<br>**Placebo:** was used, but no details
**Outcomes**	Length of ICU stay
**Notes**	This trial was identified from ClinicalTrials.gov.<br>We were able to contact Dr. Robert S. Kramer who sent the results to us (email 2016-3-23)<br>Later the data were published in ClinicalTrials.gov, see link above

Risk of bias table

Bias	Authors' judgement	Support for judgement
Random sequence generation (selection bias)	Low risk	Allocation: Randomized
Allocation concealment (selection bias)	Low risk	Masking: Double Blind (Investigator, Outcomes Assessor)
Blinding of participants and personnel (performance bias)	Low risk	Masking: Double Blind (Investigator, Outcomes Assessor)
Blinding of outcome assessment (detection bias)	Low risk	Masking: Double Blind (Investigator, Outcomes Assessor)
Incomplete outcome data (attrition bias)	Low risk	

Ebade 2014

**Methods**	Controlled trial, July 2010 to December 2013<br><br>http://www.ejca.eg.net/article.asp?issn=1687-9090;year=2014;volume=8;issue=2;spage=59;epage=65;aulast=Ebade
**Participants**	Egypt, CABG patients, 29 M / 11 F; mean age: 55 yr (SD 10 yr); 20 vit C / 20 control.<br><br>**Inclusion:** patients assigned for elective CABG surgery with cardiopulmonary bypass (CPB) were enrolled in the study.<br><br>**Exclusion:** renal or hepatic dysfunction, known hypersensitivity to the studied drugs, COPD, preoperative AF, pacemaker, class I and III antiarrhythmic agents or digoxin, any degree of AV block
**Interventions**	**Vit C before the operation:**<br>**Dose:** 2 g<br>**Method:** iv, but not explicitly stated; "after induction of anesthesia" implies iv administration<br>**Timing:** "after induction of anesthesia"<br>**Vit C after operation:**<br>**Dose:** 3 g/d "1 g every 8 h daily until the fifth post-operative day" (p 60)<br>**Method:** iv, but not explicitly stated. "Control group receiving saline infusion" suggests that vitamin C also was administered by infusion<br>**Duration:** 5 d<br>**Placebo** saline infusion
**Outcomes**	Length of ICU stay<br>Length of mechanical ventilation
**Notes**	Very poorly reported study. In sensitivity analysis we excluded the study.<br>We tried to contact Dr. Ebade to ask for the details of their methods (2015-10-30, 2015-11-1, 2015-11-18, 2015-11-18, 2015-11-23 and 2019-1-9), but we were did not get any response.<br>Ebade reported ICU duration in vitamin C group 69.3 (SD 9) hr and in control group 76.2 (SD 11.6) hr.<br>Calculation of the P-value with the Taylor series formula from the reported mean and SD-values gives P = 0.03.<br>However, Ebade reported P = 0.006 (Mann Whitney) for the effect of vitamin C on ICU stay. We used this P = 0.006 to calculate the consistent SE(log(RoM)), see Supplementary file 2.

Risk of bias table

Bias	Authors' judgement	Support for judgement
Random sequence generation (selection bias)	Unclear risk	"divided into three equal groups" (p 60) without any description of the method, alternative allocation?
Allocation concealment (selection bias)	Unclear risk	Not described
Blinding of participants and personnel (performance bias)	Unclear risk	Not described
Blinding of outcome assessment (detection bias)	Unclear risk	Not described
Incomplete outcome data (attrition bias)	Unclear risk	No description whether the 40 reported patients equals the number of randomized participants, no flow diagram

Eslami 2007

**Methods**	Randomised trial, December 2003 to August 2005<br>http://www.ncbi.nlm.nih.gov/pmc/articles/PMC1995047<br>http://www.ncbi.nlm.nih.gov/pubmed/17948074
**Participants**	Iran, CABG patients, 67 M / 33 F, mean age: 61 yr (SD 8 yr), 50 vit C / 50 control.<br><br>**Inclusion:** Patients scheduled to undergo isolated CABG; age >50 yr and treatment with beta-blockers for a target heart rate of about 60–70 bpm, at least 1 week before surgery.<br><br>**Exclusion:** a history of AF, medication with class I and III antiarrhythmic agents or digoxin, a permanent or temporary pacemaker, any degree of AV block, bradycardia, end stage renal disease, severe pulmonary disease, severe hepatic disease.
**Interventions**	**Vit C before the operation:**<br>**Dose:** 2 g "2 g of effervescent ascorbic acid tablets on the night before surgery,"<br>**Method:** po<br>**Timing:** "night before surgery"<br>**Vit C after operation:**<br>**Dose:** 2 g/d "1-g doses twice daily"<br>**Method:** po<br>**Duration:** 5 d<br>**Placebo:** No formal placebo, but the patients received many medicines and it is unlikely that they identified vitamin C among all the other administered drugs, see below. We classify that all the other drugs serve as a functional placebo to vitamin C.
**Outcomes**	Length of ICU stay
**Notes**	Funding: “The study was performed as a thesis of cardiology degree and it was supported by Tehran University of medical sciences as a survey project.” (email 2015-4-11)<br><br>Additional information was received by email from Mehdi Mousavi on 2015-4-19, see below.

Risk of bias table

Bias	Authors' judgement	Support for judgement
Random sequence generation (selection bias)	Low risk	"randomized trial"... "patients were randomly assigned to..." (p 269).<br><br>"The randomization was done with block randomization ... Randomization was done by 1 investigator blinded to the drugs therapy ... It was done with a table of 4 cell block randomization" (email 2015-4-19).
Allocation concealment (selection bias)	Low risk	See above.
Blinding of participants and personnel (performance bias)	Low risk	"The surgeons were blinded. Ascorbic acid prescription and randomization was done by me, blinded to the results of holter and follow up and holter recordings were red by Dr.<br><br>Eslami who was blinded to everything... A patient who is a candidate for cardiac surgery might take many medications, usually including aspirin, nitrates, statins, possibly ACE inhibitors or ARBs etc., and as the design of our study beta blocker prescription was done to both group, thus 2 groups were receiving lots of drugs and including placebo or not including it in the regimen might not have a serious effect on result of holter monitoring that is an objective observation. Other drugs could work as placebo for control group!" (email 2015-4-19).<br><br>In our authors' judgement, we do not consider that the findings are biased by the lack of formal placebo in the placebo group.
Blinding of outcome assessment (detection bias)	Low risk	see above
Incomplete outcome data (attrition bias)	Low risk	"There was no drop out. The study was in-hospital and thus we were able to follow all included patients" (email 2015-4-19).

Fowler 2014

**Methods**	Double-blind randomized placebo-controlled trial<br>https://doi.org/10.1186/1479-5876-12-32<br>https://www.ncbi.nlm.nih.gov/pmc/articles/PMC3937164/
**Participants**	USA, severe sepsis in the ICU, 13 M / 11 F; age range 30 to 92 yr; 18 vit C / 8 control.<br><br>**Inclusion:** 1) Presence of a systemic inflammatory response 2) Suspected or proven infection, and 3) Presence of sepsis induced organ dysfunction. If these three criteria were met within 48 hours of ICU admission, informed consent was obtained from family members of patients deemed eligible for the study<br><br>**Exclusion:** Over one year, 35 patients were screened and 26 patients were enrolled. Reasons for exclusion: (a) 3 patients had terminal cancer and were not expected to survive 24 hours (b) informed consent could not be obtained for 2 homeless septic patients (c) family remembers refused consent in 4 patients.
**Interventions**	**Vit C dosage:**<br>**Dose:** Low: 0.05 g/kg/24 h and High 0.2 g/kg/24 h, which means 3.5 g/day and 14 g/day, respectively, for a 70 kg person<br>**Method:** iv<br>**Timing:** "every six hours"<br>**Duration:** 4 d<br>**Placebo** 5% dextrose and water: "Ascorbic acid or placebo solutions were prepared in matching volumes with amber shrouding for light protection and to preserve the blind." (p 2)
**Outcomes**	Length of ICU stay
**Notes**	

Risk of bias table

Bias	Authors' judgement	Support for judgement
Random sequence generation (selection bias)	Low risk	"in a 1:1:1 ratio using a randomization scheme generated by using Research Randomizer" (p 2)
Allocation concealment (selection bias)	Low risk	"The study blind was established and maintained by the VCU Investigational Pharmacy Department where the study drug was prepared, hooded, and dispensed." (p 2)
Blinding of participants and personnel (performance bias)	Low risk	"The study blind was established and maintained by the VCU Investigational Pharmacy Department where the study drug was prepared, hooded, and dispensed." (p 2)<br><br>"Ascorbic acid or placebo solutions were prepared in matching volumes with amber shrouding for light protection and to preserve the blind."(p 2)
Blinding of outcome assessment (detection bias)	Low risk	"The study blind was established and maintained by the VCU Investigational Pharmacy Department where the study drug was prepared, hooded, and dispensed." (p 2)
Incomplete outcome data (attrition bias)	Low risk	One patient in the vitamin C group was withdrawn by family members and transferred to another institution. Another was withdrawn after hemophagocytic syndrome plus sepsis was recognised. These two patients are not included in the analysis. (p 4)

Mirmohammadsadeghi 2018

**Methods**	Double-blind quasi-randomized trial<br>https://doi.org/10.1532/hsf.1938<br>https://www.ncbi.nlm.nih.gov/pubmed/30311896
**Participants**	Iran, CABG patients, 244 M / 70 F; mean age: vitamin C 62 yr, placebo 63 yr; 160 vit C / 154 control.<br><br>**Inclusion:** patients undergoing CABG surgery alone with preoperative sinus rhythm.<br><br>**Exclusion:** complex surgery, emergency surgery, history of cardiac arrhythmia or antiarrhythmic drugs, and severe renal or hepatic failure
**Interventions**	**Vit C before the operation:**<br>**Dose:** 2 g<br>**Method:** iv<br>**Timing:** "two grams vitamin C intravenously (IV) 24 hours preoperatively"<br>**Vit C after operation:**<br>**Dose:** 1 g/d<br>**Method:** "postoperatively 500 mg every 12 hours IV for 48 hours in ICU, and 500 mg every 12 hours PO for 48 hours in ward"<br>We classify this as intravenous administration, although oral administration was used after 2 days.<br>**Duration:** 4 d<br>**Control** nothing
**Outcomes**	Length of ICU stay
**Notes**	The authors published P = 0.39 (Mann Whitney) for the effect of vitamin C on the ICU stay. We used this to calculate the consistent SE(log(RoM)), see Supplementary file 2. We asked for the SD values for the groups and they were sent to us: vitamin C group ICU duration 50.41 (SD 22.07) days and control group ICU duration 52.55 (SD 22.00) days.<br><br>We were able to contact Dr. Mirmohammadsadeghi and he kindly gave more information of the study (2019-1-26), see above and below.

Risk of bias table

Bias	Authors' judgement	Support for judgement
Random sequence generation (selection bias)	Low risk	quasi-randomized "Random assignment was performed by even and odd patient code." (p E416). Table 1 shows that the groups are balanced by age, gender, mean TSH level, ejection fraction
Allocation concealment (selection bias)	Low risk	"double-blinded" (p E415)
Blinding of participants and personnel (performance bias)	Low risk	"the patíent and the following nurses were not aware of the patient group; also the prescribing and following nurses were different persons." (p E415)<br><br>"Physicians were not aware of the groups." (email 2019-1-26).
Blinding of outcome assessment (detection bias)	Low risk	"the patíent and the following nurses were not aware of the patient group; also the prescribing and following nurses were different persons." (p E415) "Physicians were not aware of the groups." (email 2019-1-26).
Incomplete outcome data (attrition bias)	Unclear risk	"The patients were randomized to two 165 patients. 11 patients were dropped from case group and 5 patients were dropped from control group" (email 2019-1-26). Although the difference between 11 and 5 seems large in percentage terms, we do not consider that such a small absolute difference is likely to bias the negative results

Papoulidis 2011

**Methods**	Randomised placebo-controlled trial, December 2006 to March 2009<br>http://dx.doi.org/10.1510/icvts.2010.240473<br>http://www.ncbi.nlm.nih.gov/pubmed/21098510
**Participants**	Greece, CABG patients, 120 M / 50 F, mean age: vitamin C 73 yr (SD 7 yr), placebo 71 yr (SD 7 yr); 85 vit C / 85 placebo.<br><br>**Inclusion:** Patients scheduled to undergo elective isolated CABG. All patients were NYHA class III and IV and also under b-blocker therapy<br><br>**Exclusion:** <65 yr, preoperative AF, hyperoxaluria, pacemaker, severe renal or hepatic failure, medication with class I and III antiarrhythmic agents or digoxin, any degree of AV block or bradycardia with a HR <50 bpm, severe pulmonary disease, enlarged left atrium (LA diameter >4.4 cm).
**Interventions**	**Vit C before the operation:**<br>**Dose:** 2 g<br>**Method:** iv<br>**Timing:** "3 h prior the initiation of CPB"<br>**Vit C after operation:**<br>**Dose:** 1 g/d "500 mg twice a day"<br>**Method:** iv<br>**Duration:** 5 d<br>**Placebo:** intravenous administration of 0.9% saline. "Same amount and at the same time" (email).
**Outcomes**	Length of ICU stay
**Notes**	Funding: “It was more like a self funding.” (email 2015-5-15)<br>Additional information was received by emails from Pavlos Papoulidis (2015-5-15 and 2015-6-2), see above and below. "It was more like a self funding" (email).<br>Papolidis reported ICU duration in vitamin C group 1.6 (SD 0.9) d and in control group 2.1 (SD 1.1) d.<br>Calculation of the P-value with the Taylor series formula from the reported mean and SD-values gives P = 0.0011. Papoulidis reported P = 0.05 (Mann Whitney) for the effect of vitamin C on ICU stay. We used this P = 0.05 to calculate the consistent SE(log(RoM)), see Supplementary file 2.

Risk of bias table

Bias	Authors' judgement	Support for judgement
Random sequence generation (selection bias)	Low risk	"The initial random assignment was by flipping a coin ... to have an equal sample size, we reevaluated our randomization protocol and using a random generator ..." (p 122).
Allocation concealment (selection bias)	Low risk	"During the randomization stage, patients and physicians were not aware of the group to which the participants were allocated" (email 2015-6-2)
Blinding of participants and personnel (performance bias)	Low risk	"During the study, patients and physicians in charge of the treatment, surgeons carrying out the operation, and the physicians interpreting the ECG recordings were all blinded of the study group" (email 2015-6-2)
Blinding of outcome assessment (detection bias)	Low risk	See above
Incomplete outcome data (attrition bias)	Low risk	"No dropouts/withdrawals" (email 2015-5-15)

Sadeghpour 2015   Not included in the analyses, see reasons below

**Methods**	Double-blind randomised placebo-controlled trial.<br>http://www.ncbi.nlm.nih.gov/pubmed/25789244<br>http://www.ncbi.nlm.nih.gov/pmc/articles/PMC4350190<br><br>Duplicate publication in Farsi (Persian):<br>Moludi J , Keshavarz S , Pakzad R , Sedghi N , Sadeghi T and Alimoradi F.<br>Effect of vitamin C supplementation in the prevention of atrial fibrillation. [Persian]<br>Tehran University Medical Journal, 2016, 73(11), 791<br>http://tumj.tums.ac.ir/browse.php?a_code=A-10-25-5413&sid=1&slc_lang=en
**Participants**	Iran, CABG or valvular surgery patients, 191 M / 99 F, mean age: vitamin c 57 yr (SD 14 yr), placebo 54 yr (SD 14yr); 113 vit C / 177 placebo.<br><br>**Inclusion:** >18 yr with American Society of Anesthesiologists physical status class II-III and candidacy for CABG or simple congenital valvular disease surgery.<br><br>**Exclusion:** who died within the 1st postoperative day and those who had not received adequate doses of drugs according to our protocol, severe complications (cardiac, respiratory or neurological) or emergency operation.
**Interventions**	**Vit C before the operation:**<br>**Dose:** 2 g<br>**Method:** iv<br>**Timing:** "immediately before surgery"<br>**Vit C after operation:**<br>**Dose:** 1 g/d<br>**Method:** po<br>**Duration:** 4 d<br>**Placebo:** "The patients in the placebo group received an equal number of identical tablets. The placebo tablets and ampoules were prepared in the same shape and size as the original" (p 2). Before surgery: "The Vit C was given in the operating room along with the other infusions by anesthesiologist technician" (email 2015-5-11).
**Outcomes**	Length of ICU stay<br>Length of mechanical ventilation
**Notes**	"We paid it by ourselves besides getting help from the Rajaei cardiovascular research center." (email 2015-10-1)<br><br>Additional information was received by email from Anita Sadeghpour (2015-5-12 and 2015-10-1), see below and above.

Although the study was reported as block randomized, the difference in the size of the groups is large and inconsistent with block randomization.

In 2015 we received a response describing that 42% of participants were excluded after randomization. The publication describes 113 vit C / 177 placebo participants which indicates that there is substantial difference in the exclusion rate between the vitamin C and placebo groups.

This is such a severe violation of the ITT principle that we exclude the trial from our analysis.

Risk of bias table

Bias	Authors' judgement	Support for judgement
Random sequence generation (selection bias)	Low risk	"The study population was randomized one day before surgery to two groups (By using www.randomizer.org) ... The method of randomization was balanced block with an allocation sequence based on a block size of eight" (p 2).<br><br>However, the sizes of the groups 113 vit C / 177 placebo are not consistent with block randomization. Therefore we exclude this study in our sensitivity analysis.
Allocation concealment (selection bias)	Low risk	"Both the patients and the hospital staff were blind to the treatment allocation" (p 2).
Blinding of participants and personnel (performance bias)	Low risk	"Both the patients and the hospital staff were blind to the treatment allocation" (p 2).<br><br>"The Vit C was given in the operating room along with the other infusions by anesthesiologist technician" (email 2015-5-12).<br><br>"The patients in the intervention group received 2 g of vitamin C ... intravenously, immediately before surgery in the operating theatre, followed by 1 g daily oral doses of the tablets for the first four postoperative days. The patients in the placebo group received an equal number of identical tablets. The placebo tablets and ampoules were prepared in the same shape and size as the original ones manufactured by the same pharmaceutical company." (p 2).
Blinding of outcome assessment (detection bias)	Low risk	See above.
Incomplete outcome data (attrition bias)	High risk	"we enrolled 500 patients but we excluded the patients who died on the first postoperative day, those who needed re operation due to technical problems and excessive bleeding, and those who had not received an adequate dose." (email 2019-1-24).<br><br>Thus, data is published of 290 participants of 500 enrolled, which means

that data of 42% of participants are missing.

This is such a severe violation of the ITT principle that we exclude the trial from our analysis.

Sarzaeem 2014

**Methods**	Double-blind randomised placebo-controlled trial, August 2012 to January 2013.<br>http://tumj.tums.ac.ir/browse.php?a_id=5853&sid=1&slc_lang=en<br>http://tumj.tums.ac.ir/browse.php?a_id=5853&slc_lang=en&sid=1&ftxt=1<br>Translation available at:<br>http://www.mv.helsinki.fi/home/hemila/T14.pdf
**Participants**	Iran, CABG patients, 118 M / 52 F, mean age: 59 yr (SD 10 yr) ; 85 vit C / 85 placebo.<br><br>**Inclusion:** Patients with coronary artery disease (in angiography) who were candidates for coronary artery bypass<br><br>**Exclusion:** >80 yr, AF before surgery; valvular heart disease, arrhythmia, or cardiac conduction block of any degree; pacemaker, chronic lung, liver, or kidney disease; other heart surgeries along with CABG, history of antiarrhythmic drug consumption, sick sinus syndrome; symptoms or history of urinary calculi, vitamin C consumption during the last 3 months.
**Interventions**	**Vit C before the operation:**<br>**Dose:** 2 g<br>**Method:** iv<br>**Timing:** "12 h before the procedure"<br>**Vit C after operation:**<br>**Dose:** 1 g/d<br>**Method:** iv<br>**Duration:** 5 d<br>**Placebo:** "patients in the control group received placebo (normal saline intravenously)" (abstract).
**Outcomes**	Length of ICU stay
**Notes**	The report was published in Farsi (Persian) with an abstract in English.<br>No reply to our emails.<br>We arranged translation of the report into English.<br>http://www.mv.helsinki.fi/home/hemila/T14.pdf<br>In table, the results were reported to one digit. The text section reports that there was a 0.49 day difference in the length of ICU stay. Therefore, to make the comparison more accurate, we adjusted the vitamin C group ICU stay to 2.51 d, keeping the placebo group value as in the table.

Risk of bias table

Bias	Authors' judgement	Support for judgement
Random sequence generation (selection bias)	Low risk	"using a table of random numbers are divided into intervention and control groups to receive placebo or vitamin C" (abstract).
Allocation concealment (selection bias)	Low risk	Double-blind implies allocation concealment
Blinding of participants and personnel (performance bias)	Low risk	"this double-blind, parallel clinical trial" (Abstract),<br><br>"The present study was a double-blind parallel group clinical trial, because neither the patients nor the health care workers were aware of the medications in the infusions" (translation)
Blinding of outcome assessment (detection bias)	Low risk	See above.
Incomplete outcome data (attrition bias)	Unclear risk	No description whether the 170 reported patients equals the number of randomized participants, no flow diagram

Tanaka 2000

**Methods**	Quasi-randomized trial, December 1992 to December 1997<br>https://doi.org/10.1001/archsurg.135.3.326<br>https://www.ncbi.nlm.nih.gov/pubmed/10722036
**Participants**	Japan, Burn patients, 25 M / 12 F; mean age: 45 yr (SD 22 yr); 19 vit C / 18 control.<br><br>**Inclusion:** "older than 16 years; thermal injury within 2 hours before admission; burn covering greater than 30% of total body surface area"<br><br>**Exclusion: "**preexisting hepatic, respiratory, cardiac, or renal dysfunction; and preexisting coagulopathy."
**Interventions**	**Vit C dosage:**<br>**Dose:** "66 mg/kg per hour" corresponds to 55.4 g/day for a 70 kg patient<br>**Method:** iv<br>**Timing:** "24-hour study period" (p 327) Thus, vitamin C was administered for just 1 day.<br>**Placebo** no placebo. "The control group did not receive the ascorbic acid infusion", but "The administered volume of ascorbic acid was included in the 24-hour fluid intake calculations." (p 327)
**Outcomes**	Length of mechanical ventilation
**Notes**	

Risk of bias table

Bias	Authors' judgement	Support for judgement
Random sequence generation (selection bias)	Low risk	Quasi-randomization: "Randomization was performed according to the month of admission" (p 327) It is unlikely that randomization of burn patients according to the month of admission causes systematic bias. There were no baseline difference in age, sex, weight, kinds of burns, areas of burns, thickness of burns, or in the rate of inhalation injuries (Table 1 and Fig 1).
Allocation concealment (selection bias)	Low risk	Quasi-randomization: "Randomization was performed according to the month of admission" (p 327) It is unlikely that randomization of burn patients according to the month of admission causes systematic bias. There were no baseline difference in age, sex, weight, kinds of burns, areas of burns, thickness of burns, or in the rate of inhalation injuries (Table 1 and Fig 1).
Blinding of participants and personnel (performance bias)	Low risk	"The administered volume of ascorbic acid was included in the 24-hour fluid intake calculations." (p 327) This means that patients had same amount of fluids in both groups. In addition, it is highly unlikely that patient with a burn covering greater than 30% TBSA would observe their treatments.
Blinding of outcome assessment (detection bias)	Unclear risk	Not described
Incomplete outcome data (attrition bias)	Unclear risk	No description whether the 37 reported patients equals the number of allocated participants, no flow diagram

Zabet 2016

**Methods**	Double-blind randomized trial, September 2014 to January 2016<br>https://dx.doi.org/10.4103/2279-042X.179569<br>https://www.ncbi.nlm.nih.gov/pmc/articles/PMC4843590<br>https://www.ncbi.nlm.nih.gov/pubmed/27162802
**Participants**	Iran, Sepsis patients, 21 M / 7 F; mean age: 64 yr (SD 16 yr); 14 vit C / 14 control.<br><br>**Inclusion:** (18–65-year-old) surgical critically ill patients with diagnosis of septic shock who needed a vasopressor drug to maintain mean arterial pressure >65 mmHg despite adequate fluid resuscitation were recruited<br><br>**Exclusion:** Not described
**Interventions**	**Vit C at the ICU:**<br>**Dose:** "25 mg/kg intravenous ascorbic acid every 6 h for 72 hours" corresponds to 7 g/day for a 70 kg patient<br>**Method:** iv<br>**Duration:** 72 hours<br>**Placebo** "Patients in the placebo group received 50 ml of dextrose 5% solution as intravenous infusion"
**Outcomes**	Length of ICU stay<br>Length of mechanical ventilation
**Notes**	

Risk of bias table

Bias	Authors' judgement	Support for judgement
Random sequence generation (selection bias)	Low risk	"according to the permuted block randomization. The randomization scheme consisted of seven blocks and each block contained four patients in random order." (p 95)
Allocation concealment (selection bias)	Low risk	"The researchers, ICU nurses, and physicians were blinded regarding the intervention and the product (ascorbic acid or placebo)" (p 95)
Blinding of participants and personnel (performance bias)	Low risk	"The researchers, ICU nurses, and physicians were blinded regarding the intervention and the product (ascorbic acid or placebo)" (p 95)
Blinding of outcome assessment (detection bias)	Low risk	"The researchers, ICU nurses, and physicians were blinded regarding the intervention and the product (ascorbic acid or placebo)" (p 95)
Incomplete outcome data (attrition bias)	Low risk	No drop-outs
